# Apoptin‐Derived Peptide Alleviates Temporal Lobe Epilepsy (TLE) by Limiting Neuronal Ca^2+^ Overload Through the HSPA1A–AP2B1 Axis

**DOI:** 10.1002/cns.71193

**Published:** 2026-10-03

**Authors:** Zixiao Tan, Qilong Zhai, Jingwen Cui, Xiaoying Gao, Xingan Liu, Zhijie Fan, Yu Liu, Jiahang Sun

**Affiliations:** ^1^ Department of Neurosurgery The Second Affiliated Hospital of Harbin Medical University Harbin Heilongjiang China; ^2^ Department of Anesthesiology The Fourth Affiliated Hospital of Harbin Medical University Harbin Heilongjiang China; ^3^ Department of Pharmacology Harbin Medical University Harbin Heilongjiang PR China

**Keywords:** AP2B1, B9 (apoptin‐derived peptide), HSPA1A, temporal lobe epilepsy

## Abstract

**Aims:**

To determine whether the apoptin‐derived peptide (also named B9) reduces evoked seizure susceptibility and kainic acid (KA)‐associated cognitive and molecular abnormalities, and to define whether these effects involve the heat shock protein family A member 1A (HSPA1A)–adaptor protein complex 2 subunit beta 1 (AP2B1) pathway.

**Methods:**

The therapeutic efficacy of B9 was examined in a pentylenetetrazol (PTZ)‐induced acute seizure model and a KA‐induced temporal lobe epilepsy (TLE) model. Electroencephalographic (EEG) recordings were performed to evaluate epileptiform activity. The Barnes maze test was used to assess cognitive function in epileptic mice. Western blotting, co‐immunoprecipitation (Co‐IP), immunofluorescence (IF), and calcium imaging were used to investigate the underlying mechanism.

**Results:**

B9 reduced seizure susceptibility, attenuated epileptiform activity, and improved spatial learning and memory. HSPA1A was markedly upregulated in TLE‐related datasets and experimental models. B9 suppressed the expression of HSPA1A and disrupted its interaction with AP2B1, which restored O‐linked β‐N‐acetylglucosamine (O‐GlcNAc) modification and protein stability of AP2B1, preserved GluA2 expression, and limited neuronal Ca^2+^ overload. Knockdown of AP2B1 abolished the effects of B9 on evoked seizure susceptibility, cognitive performance, GluA2 abundance, and neuronal Ca^2+^ overload, whereas AP2B1 overexpression reproduced the reductions in PTZ‐evoked seizure susceptibility and EEG activity observed with B9.

**Conclusion:**

B9 reduces PTZ‐evoked seizure susceptibility and ameliorates KA‐associated cognitive and molecular abnormalities through modulation of the HSPA1A–AP2B1 axis. These findings support B9 as a potential therapeutic strategy for TLE.

## Graphical abstract

Epileptogenic stress upregulates HSPA1A, impairs AP2B1 O‐GlcNAcylation and stability, and promotes GluA2 loss‐associated CP‐AMPAR formation and Ca^2+^ overload. B9 attenuates epileptogenesis by targeting the HSPA1A–AP2B1 axis, restoring AP2B1 O‐GlcNAcylation and stability, preserving GluA2‐associated AMPAR regulation, and reducing pathological Ca^2+^ influx and neuronal hyperexcitability.

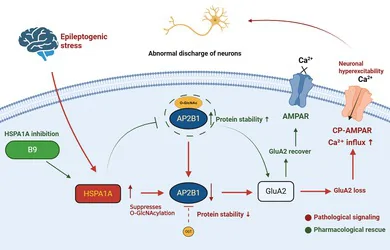

## 1 Introduction

Epilepsy, as one of the five priority neurological disorders in global public health, continues to impose a substantial burden as one of the most common chronic neurological diseases worldwide [1]. Approximately 30%–40% of patients develop drug‐resistant epilepsy, among which temporal lobe epilepsy (TLE) is the most common and refractory subtype [2]. Hippocampal sclerosis, a hallmark of TLE, involves progressive structural remodeling and persistent network hyperexcitability driven by complex molecular alterations that currently lack effective disease‐modifying pharmacological interventions [3]. Although numerous antiseizure medications are available, most provide symptomatic seizure control without modifying the underlying epileptogenic process, highlighting an urgent need for disease‐modifying pharmacological strategies targeting core drivers of neuronal hyperexcitability [4].

Pathological neuronal hyperexcitability in TLE is closely associated with synaptic receptor remodeling, disrupted calcium homeostasis, and maladaptive post‐translational signaling [5]. In particular, reduced expression of the α‐amino‐3‐hydroxy‐5‐methyl‐4‐isoxazolepropionic acid (AMPA) receptor subunit GluA2 promotes the formation of Ca^2+^‐permeable AMPA receptors (CP‐AMPARs). Reduction of the GluA2 subunit of AMPA receptors (AMPARs) leads to increased formation of calcium‐permeable AMPARs (CP‐AMPARs), thereby enhancing intracellular Ca^2+^ influx and promoting excitotoxicity during epileptogenesis [6, 7, 8]. CP‐AMPAR generation has been implicated as an early and sustained contributor to seizure susceptibility and synaptic dysfunction [6, 9, 10]. Therefore, pharmacological strategies capable of stabilizing synaptic receptor composition and limiting pathological Ca^2+^ overload may represent promising mechanism‐directed approaches in epilepsy, although disease‐modifying efficacy requires evidence that the development or long‐term course of epilepsy is altered. However, the upstream stress‐associated mechanisms driving CP‐AMPAR remodeling during epileptogenesis remain poorly defined and are not effectively targeted by current therapies.

Heat shock protein 70 (HSP70) family members function as molecular chaperones that regulate protein folding, stability, and stress responses. Among them, heat shock protein family A member 1A (HSPA1A) represents the major stress‐inducible cytosolic isoform in neurons and is strongly upregulated under cellular stress conditions [11]. Previous studies have suggested context‐dependent roles of HSP70 proteins in neurological diseases: HSPA1A exhibits protective effects in certain neurodegenerative disorders such as polyglutamine diseases and Parkinson's disease [12, 13], whereas excessive or sustained activation has been associated with disease progression in Alzheimer's disease [14]. Importantly, most previous studies have examined HSP70 family activity without distinguishing individual isoforms, and mechanistic investigations focusing specifically on HSPA1A in neuronal signaling remain limited. In the context of epilepsy, only one report suggested that Hsp70 may facilitate seizure onset by promoting degradation of the Kv4–KChIP4a complex during early epileptogenesis [15]. However, whether the HSPA1A isoform specifically contributes to synaptic remodeling and epilepsy progression has not been systematically investigated. Therefore, the mechanistic role of HSPA1A in epileptogenesis and its potential as a therapeutic target remain to be clarified.

Apoptin‐derived peptide (ADP), also known as B9, is a polypeptide drug composed of active fragments of apoptin and a trans‐activator of transcription (TAT) cell‐penetrating sequence [16, 17]. B9 was originally developed as an anti‐cancer agent, particularly in glioblastoma, where it suppresses HSP70 transcription through interaction with heat‐shock elements (HSEs) within the HSP70 promoter [18]. In addition, B9 has been shown to overcome drug resistance in malignancies through stress‐signaling modulation [19]. In our previous glioma orthotopic xenograft model, B9 administration was associated with a reduction in spontaneous epileptiform activity and seizure‐like behaviors, suggesting its potential anti‐seizure activity.

In this study, we examined whether B9 reduces pentylenetetrazol (PTZ)‐evoked seizure susceptibility and ameliorates kainic acid (KA)‐associated cognitive and molecular abnormalities. Mechanistically, B9 inhibits the expression of HSPA1A, restores synaptic receptor homeostasis, and limits pathological calcium overload driven by stress‐associated signaling. Collectively, our findings support further investigation of B9 as a potential therapeutic candidate for TLE.

## 2 Materials and Methods

### 2.1 Human Brain Tissue Samples

Human brain tissues were obtained from patients who underwent clinically indicated neurosurgical procedures at the Second Affiliated Hospital of Harbin Medical University between 2024 and 2026. The collection and use of human brain tissues were approved by the Ethics Committee of the Second Affiliated Hospital of Harbin Medical University (approval no. KY2024‐125), and all procedures involving human samples were conducted in accordance with the Declaration of Helsinki. Written informed consent was obtained from all participants or their legal guardians before sample collection. All tissue samples and associated clinical data were de‐identified before analysis. The TLE samples consisted of sclerotic hippocampal tissues resected during clinically indicated surgery from patients with drug‐resistant TLE diagnosed by clinical evaluation, preoperative video‐EEG and MRI, and postoperative histopathological confirmation of hippocampal sclerosis. Six TLE and six trauma‐control specimens were used for western blotting, and sclerotic hippocampal specimens from three additional, non‐overlapping patients with drug‐resistant TLE were used for CoIP–MS.

Control tissues were obtained from patients undergoing surgery for traumatic injury involving the temporal lobe who had no history of epilepsy. These specimens consisted of injured temporal cortical tissues that required removal as part of clinical treatment; no brain tissue was removed solely for research purposes. All tissues were snap‐frozen in liquid nitrogen immediately after surgical resection and stored in liquid nitrogen until analysis. The demographic and clinical characteristics of the participants are provided in Table S2.

### 2.2 KA Model, B9 Administration, and Tissue Collection

Adult male C57BL/6 mice (6–8 weeks old, approximately 23 g) were used for all in vivo experiments. Only male mice were included to reduce potential variability associated with estrous cycle‐dependent changes in seizure burden and pharmacological responses [20, 21]. For KA modeling, mice were anesthetized with isoflurane and received an intracerebroventricular injection of KA (1 μL, 0.25 mg/mL; Guangzhou Shuopu Biotechnology Co. Ltd.) at the following coordinates relative to bregma: AP, −0.7 mm; ML, ±0.1 mm; and DV, −1.9 mm from the dura. KA was dissolved in phosphate‐buffered saline (PBS) and filtered through a 0.22‐μm membrane before use. Sham mice received an equivalent volume of sterile PBS. Seizure severity was assessed using the Racine scale (Table S1), and mice reaching Stage IV or V were considered successfully modeled.

B9 was synthesized by Peptide Biological (Zhejiang, China), with the following amino acid sequence:

H‐Met‐Gly‐Arg‐Lys‐Lys‐Arg‐Arg‐Gln‐Arg‐Arg‐Arg‐Leu‐Lys‐Pro‐Pro‐Ser‐Lys‐Lys‐Arg‐Ile‐Arg‐Ile‐Gly‐Ile‐Ala‐Gly‐Ile‐Thr‐Ile‐Thr‐Leu‐Ser‐Leu‐Arg‐Pro‐Arg‐Thr‐Ala‐Lys‐Arg‐Arg‐Ile‐Arg‐Leu‐OH. B9 was dissolved in sterile PBS, filtered through a 0.22‐μm membrane, and administered by tail vein injection in a volume of 200 μL. KA injection was designated as Day 0. In the dose‐ranging experiment, mice received B9 at 1, 3, or 6 mg/kg/day or PBS once daily on Days 2–8. Based on the balance between behavioral efficacy and tolerability, 3 mg/kg/day was selected for subsequent experiments.

For the untreated KA time‐course analysis, hippocampal tissues were collected from separate cohorts on Days 3, 7, 14, and 30 after KA injection. For the B9 treatment‐duration analysis, KA‐treated mice received B9 (3 mg/kg/day) once daily from Day 2 until tissue collection on Day 3, 7, 14, or 30. A single KA comparator cohort received PBS on the same schedule and was collected on Day 7. The sham comparator cohort was collected at the same endpoint. These groups were analyzed as independent experimental groups rather than as a factorial time‐by‐treatment design.

### 2.3 Culture of Primary Neurons

Hippocampal tissues were carefully dissected from E18 Sprague–Dawley rat embryos of both sexes and immediately transferred into ice‐cold D‐Hank's balanced salt solution (HBSS; Solarbio, Beijing, China). The tissues were cut into small pieces and enzymatically digested with 0.125% trypsin (Gibco) at 37°C for 10 min. After dissociation, cells were plated onto poly‐L‐lysine (Sigma‐Aldrich)‐coated coverslips and maintained in Dulbecco's modified Eagle's medium supplemented with 10% fetal bovine serum (FBS) and 1% penicillin/streptomycin (P/S). After 4 h, the culture medium was switched to Neurobasal medium containing 2% B27, 1% P/S, and 1% L‐glutamine (Gibco). Neurons were cultured at 37°C in a humidified atmosphere with 5% CO_2_, and half of the medium was renewed every 3 days. Rat primary hippocampal neurons were used for in vitro mechanistic assays because they provide robust neurite development and stable synaptic protein expression, and all treatment comparisons were performed within the same culture system.

### 2.4 Bioinformatics Analysis

The processed gene‐expression matrix of GSE256068 was obtained from the NCBI Gene Expression Omnibus database. TLE‐HS (*n* = 64) and ControlCortex (*n* = 14) samples were included, whereas other diagnostic groups were excluded. According to the GEO processing record, the data were normalized using the trimmed mean of *M*‐values method and processed with voom; no additional normalization or transformation was performed. Differential expression between TLE‐HS and Control Cortex was analyzed using limma. Differentially expressed genes were defined as |log_2_ fold change| > 0.5 and nominal *p* < 0.05, with Benjamini‐Hochberg‐adjusted *p* values also reported. Gene Ontology enrichment was performed using clusterProfiler, with adjusted *p* < 0.05 and *q* < 0.20 as the thresholds. Kyoto Encyclopedia of Genes and Genomes enrichment was performed separately for upregulated and downregulated genes using gprofiler2, with g *p* < 0.05 considered significant.

### 2.5 In Vivo Adaptor Protein Complex 2 Subunit Beta 1 (AP2B1) Knockdown and Overexpression

AAV9‐AP2B1 (1 × 10^12^ vg/mL) or AAV‐NC was used for AP2B1 overexpression, whereas sh‐AP2B1 lentivirus (1 × 10^8^ TU/mL) or sh‐NC was used for AP2B1 knockdown. Viral vectors were bilaterally injected into the hippocampus at the following coordinates relative to bregma: AP, −2.0 mm; ML, ±1.5 mm; and DV, −1.5 mm from the dura. Each hemisphere received 0.5 μL at 0.05 μL/min, and the needle was left in place for 5 min. Subsequent experiments were performed 2–3 weeks after viral injection.

Separate cohorts were used for the cumulative PTZ challenge, acute PTZ/EEG recording, and KA‐associated experiments. Mice in the PTZ cohorts received B9 (3 mg/kg/day) or PBS on Days 1–7 and were tested on Day 8 without KA administration. In the KA cohorts, KA injection was designated as Day 0, followed by B9 or PBS administration on Days 2–8. Hippocampal tissues were collected on Day 9. Barnes maze testing was conducted on Days 9–18 without further B9 or PBS administration.

### 2.6 In Vivo Electroencephalography (EEG) Recording

The acute PTZ/EEG experiment was performed in a separate cohort without KA exposure. Mice received B9 (3 mg/kg/day, i.v.) or PBS once daily on Days 1–7, followed by EEG assessment on Day 8. Under isoflurane anesthesia, a positive recording electrode was stereotaxically inserted unilaterally into the hippocampus (AP, −2.20 to −2.22 mm; ML, +1.35 mm; DV, −1.80 mm). A negative reference electrode was placed on the skull surface, and a ground electrode was attached to the tail. EEG recording began 24 h after electrode placement. Awake mice underwent a 15‐min baseline recording, followed by PTZ administration (80 mg/kg, i.p.) and a 15‐min post‐PTZ recording. EEG signals were acquired using the BL‐420S Biological Function Experiment System at a sampling rate of 50 Hz.

For all mice, the hippocampal EEG signal from the same predefined 0–10‐min post‐PTZ interval was processed to generate an amplitude‐envelope trace. The temporal mean of the envelope amplitude was calculated as one value per mouse (μV; *n* = 6 per group). This parameter represents the average magnitude of acute PTZ‐evoked hippocampal EEG activity but does not independently measure seizure frequency, duration, or spontaneous recurrent seizures.

### 2.7 Cumulative Pentylenetetrazol (PTZ) Challenge Test

The cumulative PTZ challenge was performed in a cohort separate from the KA/Barnes maze cohort, and these mice did not receive KA. Mice were randomly assigned to the PBS + PTZ and B9 + PTZ groups. B9 was administered by tail vein injection at 3 mg/kg/day in a volume of 200 μL once daily for seven consecutive days (Days 1–7), whereas control mice received an equal volume of sterile PBS according to the same schedule. On Day 8, mice underwent the PTZ challenge. The 7‐day treatment duration was prespecified as a short‐course repeated‐dose regimen to evaluate B9 after repeated systemic administration rather than after a single acute dose and was aligned with the treatment window used for the KA‐associated behavioral experiments. The PTZ challenge was scheduled on Day 8, approximately 24 h after the final B9 or PBS administration, so that no treatment injection was administered during the challenge session, thereby minimizing acute injection‐ and handling‐related influences on behavioral seizure scoring. PTZ (Shanghai Aladdin Bio‐Chem Technology Co. Ltd.) was freshly prepared in sterile normal saline, filtered through a 0.22‐μm membrane, and administered intraperitoneally at 10 mg/kg per injection in a volume of 200 μL every 10 min within a single experimental session until each mouse first reached Racine Stage IV or V. Seizure severity was scored after each injection using the Racine scale (0–VI). The primary outcome was the number of PTZ injections required to reach the predefined Racine Stage IV/V endpoint. This acute PTZ challenge was used to quantify susceptibility to pharmacologically evoked seizures under γ‐aminobutyric acid type A (GABA_A_) receptor‐mediated disinhibition and was not intended to assess spontaneous seizure generation or epileptogenesis.

### 2.8 Barnes Maze Test

The Barnes maze consisted of a circular platform with evenly spaced holes around the perimeter, one of which led to an escape box. The target hole and distal visual cues remained fixed throughout the experiment.

KA injection was designated as Day 0. B9 or PBS was administered once daily on Days 2–8. Barnes maze testing began on Day 9, approximately 24 h after the final administration, and no treatment was given during the testing period.

On Day 9, each mouse was placed in the escape box for 15 s and then underwent a 200‐s habituation and initial acquisition trial from the center of the platform. On Days 10–14, mice underwent two 180‐s acquisition trials per day, with the starting orientation alternated between trials. Each trial ended when the mouse entered the escape box. Mice that failed to locate the target hole within the allotted time were gently guided to the escape box and allowed to remain there for 15 s. No maze training was performed on Days 15–17.

On Day 18, the escape box was removed or made inaccessible, and each mouse underwent a 90‐s probe trial. All trials were video‐recorded and analyzed using the SuperMaze tracking system. Total distance traveled, latency to reach the target hole, and the number of errors before reaching the target hole were recorded.

### 2.9 Fluo‐4 Calcium Imaging

#### 2.9.1 Reagents

(1) A 2 mM Fluo‐4 acetoxymethyl ester (Fluo‐4 AM) stock solution prepared by dissolving 1 mg of Fluo‐4 AM in 442 μL dimethyl sulfoxide (DMSO); (2) Pluronic F127; (3) HBSS; and (4) 4‐(2‐hydroxyethyl)‐1‐piperazineethanesulfonic acid (HEPES)‐buffered saline containing 10 mM HEPES, 1 mM Na_2_HPO₄, 137 mM NaCl, 5 mM KCl, 1 mM CaCl_2_, 0.5 mM MgCl_2_, 5 mM glucose, and 0.1% bovine serum albumin (pH 7.4).

#### 2.9.2 Experimental Procedure

Pluronic F127 (16.5 mg) was mixed with the Fluo‐4 AM/DMSO stock solution to prevent dye aggregation in HBSS and enhance cellular loading efficiency. The Fluo‐4 AM solution was then diluted with HBSS to achieve a final concentration of 4 μM. Cells were incubated with the Fluo‐4 AM solution at 37°C for approximately 20 min. Subsequently, five volumes of HBSS supplemented with 1% FBS were added, followed by an additional incubation for 40 min. Cells were washed three times with HEPES‐buffered saline and resuspended in the same buffer to a final density of 1 × 10^5^ cells/mL. The cell suspension was further incubated at 37°C for 10 min prior to fluorescence‐based calcium imaging (excitation: 494 nm; emission: 516 nm).

### 2.10 Co‐Immunoprecipitation (Co‐IP)

Co‐IP assays were conducted using transfected human embryonic kidney 293 (HEK293) cells and mouse hippocampal tissues. Cells were rinsed three times with ice‐cold phosphate‐buffered saline (PBS) before lysis. Mouse hippocampi were homogenized by brief sonication for 10 s. Both cell lysates and tissue homogenates were prepared in lysis buffer containing 150 mM NaCl, 20 mM Tris, 1% Triton X‐100, 1% sodium deoxycholate, 10 mM ethylenediaminetetraacetic acid (EDTA), and a protease inhibitor cocktail (Roche Applied Science) at pH 8.0, followed by incubation at 4°C for 30 min.

After clarification by centrifugation at 13,000 × *g* for 10 min, the supernatants were collected for immunoprecipitation. An aliquot equivalent to 200 μg of total protein was incubated with either ANTI‐FLAG M2 Affinity Gel (Sigma) or Protein A/G PLUS‐Agarose (Santa Cruz Biotechnology) in the presence of an anti‐HSPA1A antibody (1 μg; Abcam, ab2787). Immunoprecipitation reactions were performed for 5 h or overnight at 4°C with gentle rotation. A separate fraction of each lysate was retained as the input control.

Following incubation, immunocomplexes were washed four times with Tris‐buffered saline and subsequently eluted using 0.1 M glycine hydrochloride (pH 3.5). Both input samples and immunoprecipitated proteins were analyzed by western blotting. All experiments were independently repeated at least three times.

### 2.11 Co‐Immunoprecipitation Mass Spectrometry (Co‐IP/MS) Analysis

Co‐IP/MS was performed using fresh‐frozen sclerotic hippocampal specimens from patients with drug‐resistant TLE. Fresh‐frozen hippocampal samples were homogenized on ice in lysis buffer containing 150 mM NaCl, 20 mM Tris, 1% Triton X‐100, 1% sodium deoxycholate, 10 mM EDTA, and a protease inhibitor cocktail (Roche Applied Science; pH 8.0), followed by incubation at 4°C for 30 min. Tissue lysates were clarified by centrifugation at 13,000 × *g* for 10 min, and protein concentrations were measured using a bicinchoninic acid (BCA) assay.

Equal amounts of total protein were incubated overnight at 4°C with gentle rotation with an anti‐HSPA1A antibody prebound to Protein A/G PLUS‐Agarose (Santa Cruz Biotechnology), while normal immunoglobulin G (IgG) served as a negative control. Immune complexes were washed multiple times with Tris‐buffered saline and eluted using 0.1 M glycine hydrochloride (pH 3.0). Eluted proteins were separated by sodium dodecyl sulfate–polyacrylamide gel electrophoresis (SDS–PAGE) and visualized by Coomassie Brilliant Blue staining. Protein bands were excised, subjected to in‐gel trypsin digestion, and the resulting peptides were analyzed by liquid chromatography–tandem mass spectrometry (LC–MS/MS) at a commercial proteomics facility. MS/MS spectra were searched against the UniProt human protein database using standard parameters, and peptide and protein identifications were accepted at a false discovery rate of less than 1%.

### 2.12 Cycloheximide (CHX) Treatment

To evaluate AP2B1 protein stability, N2a cells were treated with KA in the presence or absence of B9, followed by cycloheximide (CHX) to block de novo protein synthesis. Cells were collected at 0, 30, 60, and 120 min after CHX addition and lysed in buffer containing 150 mM NaCl, 20 mM Tris, 1% Triton X‐100, 1% sodium deoxycholate, 0.1% SDS, 10 mM EDTA, and a protease inhibitor cocktail (Roche Applied Science). Lysates were clarified by centrifugation at 13,000 × *g* for 10 min, and supernatants were subjected to western blot analysis.

### 2.13 Western Blot Analysis

Protein samples were separated by SDS–PAGE and transferred onto polyvinylidene fluoride membranes (Millipore). After membrane blocking, the membranes were incubated with primary antibodies against HSPA1A, AP2B1, GluA2, O‐linked N‐acetylglucosamine (O‐GlcNAc), O‐GlcNAc transferase (OGT), and glyceraldehyde‐3‐phosphate dehydrogenase (GAPDH) at 4°C overnight. After incubation with appropriate horseradish peroxidase–conjugated secondary antibodies, immunoreactive bands were detected using an enhanced chemiluminescence system (Millipore). Signal intensities were quantified with Quantity One software (Bio‐Rad) and normalized to GAPDH.

### 2.14 Quantification and Statistical Analysis

Data are presented as mean ± SEM. For animal, human tissue, and cell‐culture experiments, *n* denotes independent mice, patients, and wells, respectively. Measurements from multiple cells, fields, or tissue sections within the same experimental unit were averaged before analysis. Normality was assessed using the Shapiro–Wilk test. Normally distributed data were analyzed using unpaired two‐tailed Student's *t*‐tests or one‐ or two‐way ANOVA followed by Dunnett's test. Non‐normally distributed data were analyzed using Mann–Whitney *U* tests or Kruskal–Wallis tests followed by Dunn's test. Seizure incidence was analyzed using Fisher's exact test. Exact sample sizes and statistical tests are specified in the figure legends. Exact *p* values, relevant test statistics, degrees of freedom, and adjusted *p* values for multiple comparisons are provided in Table S3. *p* < 0.05 was considered statistically significant.

Analyses were performed using GraphPad Prism 9.0. Animals were randomly assigned to groups, and behavioral and EEG analyses were conducted blinded to group allocation. Sample sizes were based on prior experience and published studies. Data were excluded only according to predefined criteria.

## 3 Results

### 3.1 B9 Reduces PTZ‐Evoked Seizure Susceptibility and Improves KA‐Associated Cognitive Performance

In our previous orthotopic glioma model, B9 treatment significantly reduced seizure incidence, providing initial evidence that B9 may exert protective effects against pathological neuronal hyperexcitability (Figure S1). Figure 1A summarizes the treatment and assessment schedules for the cumulative PTZ challenge and acute PTZ/EEG recording, which were performed in two independent cohorts without KA exposure. In the cumulative PTZ cohort, mice did not receive KA and received PBS or B9 for 7 consecutive days before PTZ testing on Day 8. Compared with PBS‐pretreated mice, B9‐pretreated mice required significantly more incremental PTZ injections to first reach Racine Stage IV/V, indicating reduced PTZ‐evoked seizure susceptibility (Figure 1B,C). Consistently, in the independent acute PTZ/EEG cohort, which also did not receive KA, B9 pretreatment significantly reduced the mean EEG envelope amplitude during the predefined 10‐min post‐PTZ analysis window (Figure 1D,E).

**FIGURE 1:**
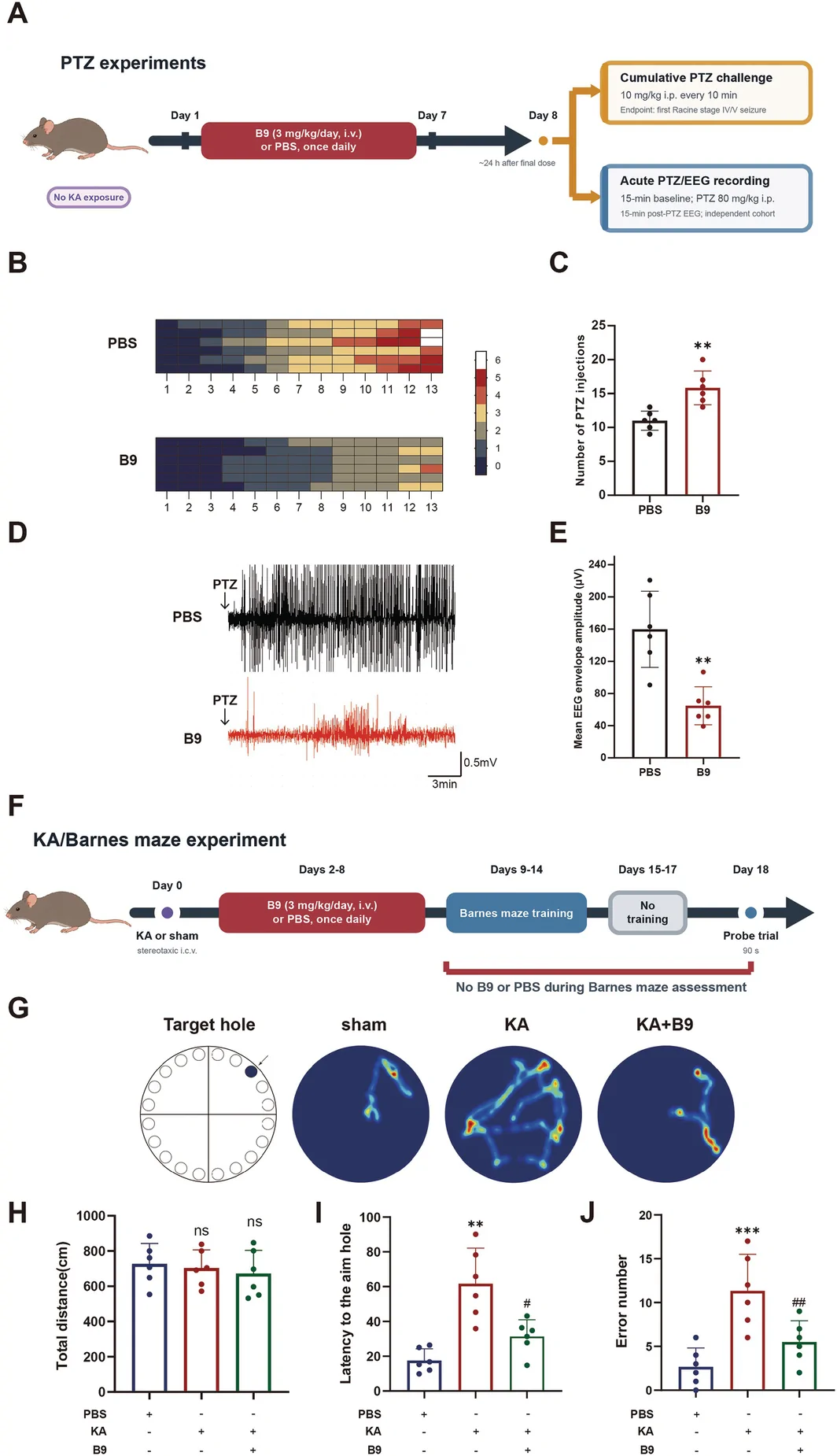
B9 reduces PTZ‐evoked seizure susceptibility and improves KA‐associated cognitive performance. (A) Experimental timelines for the independent cumulative PTZ challenge and acute PTZ/EEG cohorts. (B) Heatmap of Racine seizure scores during the cumulative PTZ challenge. (C) Number of PTZ injections required to reach Racine Stage IV/V. (D) Representative post‐PTZ EEG traces. (E) Mean post‐PTZ EEG envelope amplitude during the predefined 10‐min analysis window. For panels B–E, *n* = 6 mice per group. Data in panels C and E are presented as mean ± SEM and were analyzed using unpaired two‐tailed Student's *t*‐tests. **p < 0.01 versus the PBS group. (F) Experimental timeline for the KA/Barnes maze cohort. (G) Barnes maze schematic and representative trajectories. (H) Total distance traveled. (I) Latency to reach the target hole. (J) Number of navigation errors. For panels G–J, *n* = 6 mice per group. Panels H and J were analyzed using ordinary one‐way ANOVA followed by Dunnett's multiple‐comparisons test. Panel I was analyzed using Welch's one‐way ANOVA followed by Dunnett's T3 multiple‐comparisons test. **p < 0.01 and ***p < 0.001 versus the sham group; #*p* < 0.05 and ##*p* < 0.01 versus the KA group; ns, not significant. Exact *p* values and the corresponding test statistics and degrees of freedom, where applicable, are provided in Table S3.

Figure 1F summarizes the treatment and behavioral‐assessment schedule for the separate KA/Barnes maze cohort. In a separate KA‐treated cohort, we next assessed whether B9 ameliorated KA‐associated cognitive impairment using the Barnes maze test. Representative movement traces showed that KA‐treated mice exhibited impaired target‐directed navigation, whereas B9 treatment partially restored search performance (Figure 1G). Quantitative analysis further demonstrated that B9 significantly shortened the latency to locate the target hole and reduced the number of navigation errors compared with untreated KA mice, while total locomotor distance remained unchanged (Figure 1H–J). These findings indicate that B9 improves spatial learning and memory without affecting general motor activity. Collectively, these results show that systemic B9 treatment reduces PTZ‐evoked seizure susceptibility and ameliorates KA‐associated cognitive impairment across separate experimental cohorts.

### 3.2 B9 Suppresses Pathological HSPA1A Upregulation in Temporal Lobe Epilepsy

Given that B9 was originally developed as a stress‐targeting agent and HSPA1A represents a major target of its action [18], we next investigated whether HSPA1A is aberrantly activated in epilepsy. Differential expression analysis of the GSE256068 dataset showed that HSPA1A was significantly upregulated in TLE‐HS samples compared with ControlCortex samples (log_2_ fold change = 1.34, nominal *p* = 8.98 × 10^−6^, Benjamini‐Hochberg‐adjusted *p* = 3.11 × 10^−5^; Figure 2A). Consistently, western blot analysis showed significantly higher HSPA1A protein expression in resected sclerotic hippocampal tissues from patients with drug‐resistant TLE than in injured temporal cortical tissues from non‐epileptic trauma controls (Figure 2B,C). We next examined the temporal profile of HSPA1A in a KA‐induced epilepsy model. Western blot analysis showed that hippocampal HSPA1A expression significantly increased after KA administration and remained significantly elevated from Day 3 to Day 30 (Figure 2D,E). Importantly, B9 treatment significantly reduced HSPA1A expression throughout this period (Figure 2F,G).

**FIGURE 2:**
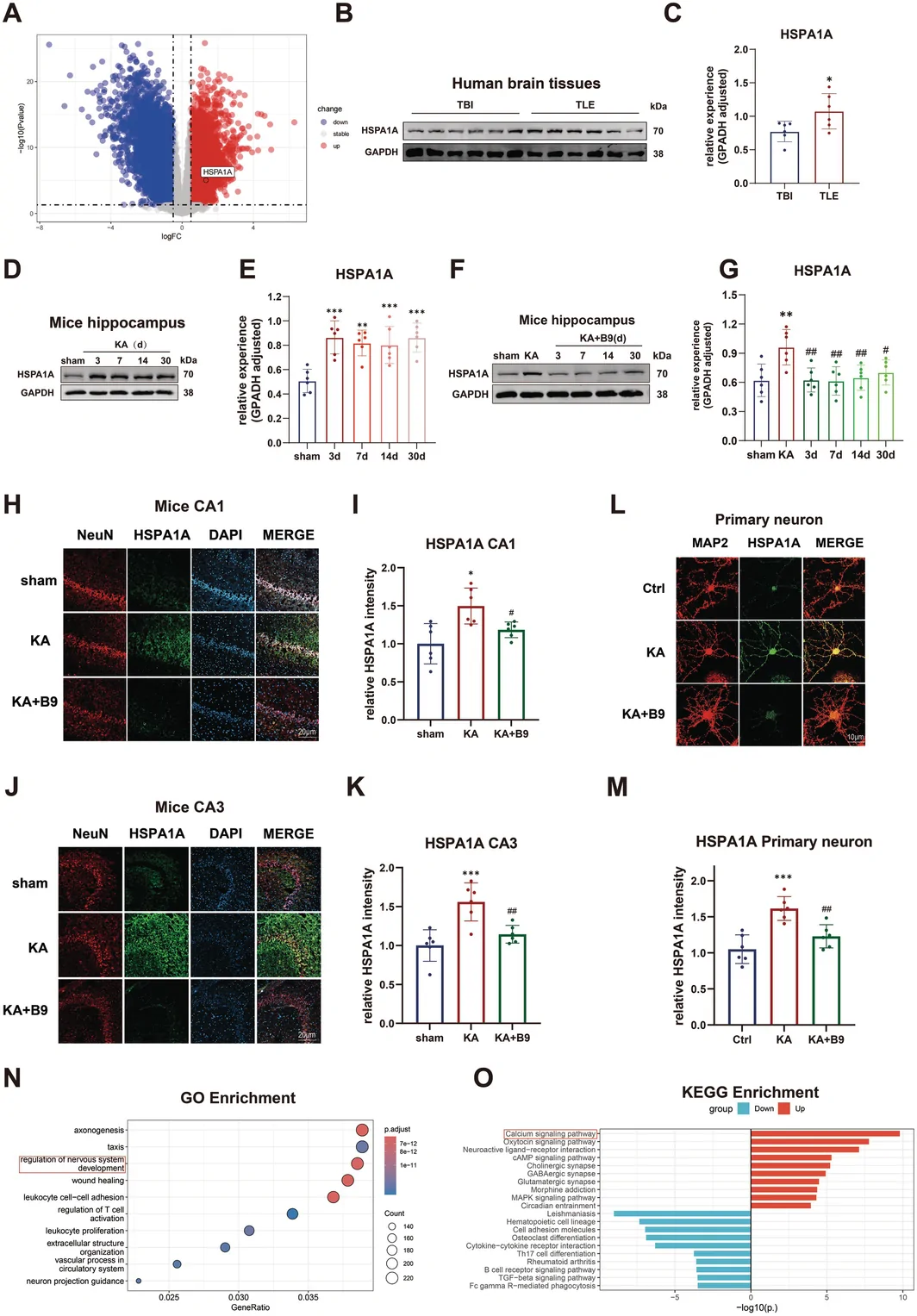
HSPA1A is persistently upregulated in epilepsy and suppressed by B9. (A) Volcano plot comparing TLE‐HS (*n* = 64) and ControlCortex (*n* = 14) samples in the GSE256068 dataset; dashed lines indicate the DEG thresholds of |log_2_ fold change| > 0.5 and nominal *p* < 0.05, and HSPA1A is indicated. (B, C) Representative western blots and densitometric quantification of HSPA1A in injured temporal cortical tissues from TBI controls and sclerotic hippocampal tissues from patients with drug‐resistant TLE. *n* = 6 independent patients per group. (D, E) Representative western blots and quantification of hippocampal HSPA1A at 3, 7, 14, and 30 days after KA injection. (F, G) Representative western blots and quantification of hippocampal HSPA1A in sham, KA, and KA + B9 mice at the indicated time points. For panels D, E, *n* = 6 mice per group at each indicated time point. For panels F, G, *n* = 6 mice in each independent experimental group. (H, I) Representative NeuN/HSPA1A immunofluorescence images and quantification of HSPA1A fluorescence intensity in the hippocampal CA1 region. (J, K) Representative NeuN/HSPA1A immunofluorescence images and quantification in the hippocampal CA3 region. *n* = 6 mice per group for panels H–K. Scale bars, 20 μm. (L, M) Representative MAP2/HSPA1A immunofluorescence images and quantification of HSPA1A fluorescence intensity in primary neurons. *n* = 6 independent wells from a single primary‐neuron culture preparation per group. Scale bar, 10 μm. (N) GO over‐representation analysis of the combined upregulated and downregulated DEGs. (O) KEGG enrichment analysis performed separately for upregulated and downregulated DEGs; the 10 most significant pathways in each direction are shown. Quantitative data are presented as mean ± SEM. Panel C was analyzed using an unpaired two‐tailed Student's *t*‐test. Panels E, G, K, and M were analyzed using ordinary one‐way ANOVA followed by Dunnett's multiple‐comparisons test. Panel I was analyzed using Welch's one‐way ANOVA followed by Dunnett's T3 multiple‐comparisons test. *p < 0.05, **p < 0.01, and ***p < 0.001 versus the corresponding TBI, sham, or control group; ^#^ *p* < 0.05 and ^##^ *p* < 0.01 versus the corresponding KA group. Exact *p* values and the corresponding test statistics and degrees of freedom, where applicable, are provided in Table S3.

Immunofluorescence staining further showed that HSPA1A fluorescence intensity was significantly increased in neuronal nuclear antigen (NeuN)‐positive neurons in both the CA1 and CA3 regions after KA treatment, whereas B9 significantly attenuated this increase (Figure 2H–K). A similar effect was observed in primary cultured neurons, in which KA significantly increased HSPA1A fluorescence intensity and B9 significantly attenuated this increase (Figure 2L,M). Finally, GO and KEGG enrichment analyses of the DEGs between TLE‐HS and ControlCortex samples identified significant enrichment of biological processes and pathways associated with neuronal development, calcium signaling, glutamatergic synapses, and other synapse‐related functions (Figure 2N,O). Notably, calcium signaling was among the most significantly enriched pathways, suggesting that Ca^2+^‐related synaptic dysregulation may be involved in HSPA1A‐associated epileptic pathology.

### 3.3 B9 Disrupts the HSPA1A–AP2B1 Interaction and Alleviates Epileptic Ca^2+^ Overload

To explore the downstream mechanism by which HSPA1A contributes to epilepsy‐associated synaptic dysfunction, we performed co‐immunoprecipitation coupled with mass spectrometry (CoIP–MS) to identify HSPA1A‐associated proteins. Candidate proteins were filtered using the following criteria: intensity ratio (HSPA1A/control) > 2, unique peptides > 2, and score > 80 (Figure 3A,D). GO and KEGG enrichment analyses showed that these HSPA1A‐associated proteins were significantly enriched in pathways related to vesicle organization, synaptic vesicle cycle (hsa: 160), and glutamatergic synapse (Figure 3B,C), suggesting a potential link between HSPA1A‐associated stress signaling and synaptic regulation. Notably, the Reactome module ‘Trafficking of GluR2‐containing AMPA receptors’ (R‐HSA‐416993) was represented in the pathway analysis. This observation provided a rationale for examining AP2B1 and GluA2 in our experimental models but was considered hypothesis‐generating rather than evidence of a causal CP‐AMPAR mechanism. We therefore integrated the candidate protein set with a GRIA2‐centered interaction network using Search Tool for the Retrieval of Interacting Genes/Proteins (STRING) analysis. Among the candidate proteins linked to GRIA2, AP2B1 showed the closest association with HSPA1A and was therefore selected for further mechanistic validation (Figure 3E). Molecular docking further supported the structural plausibility of a direct interaction between HSPA1A and AP2B1 (Figure 3F).

**FIGURE 3:**
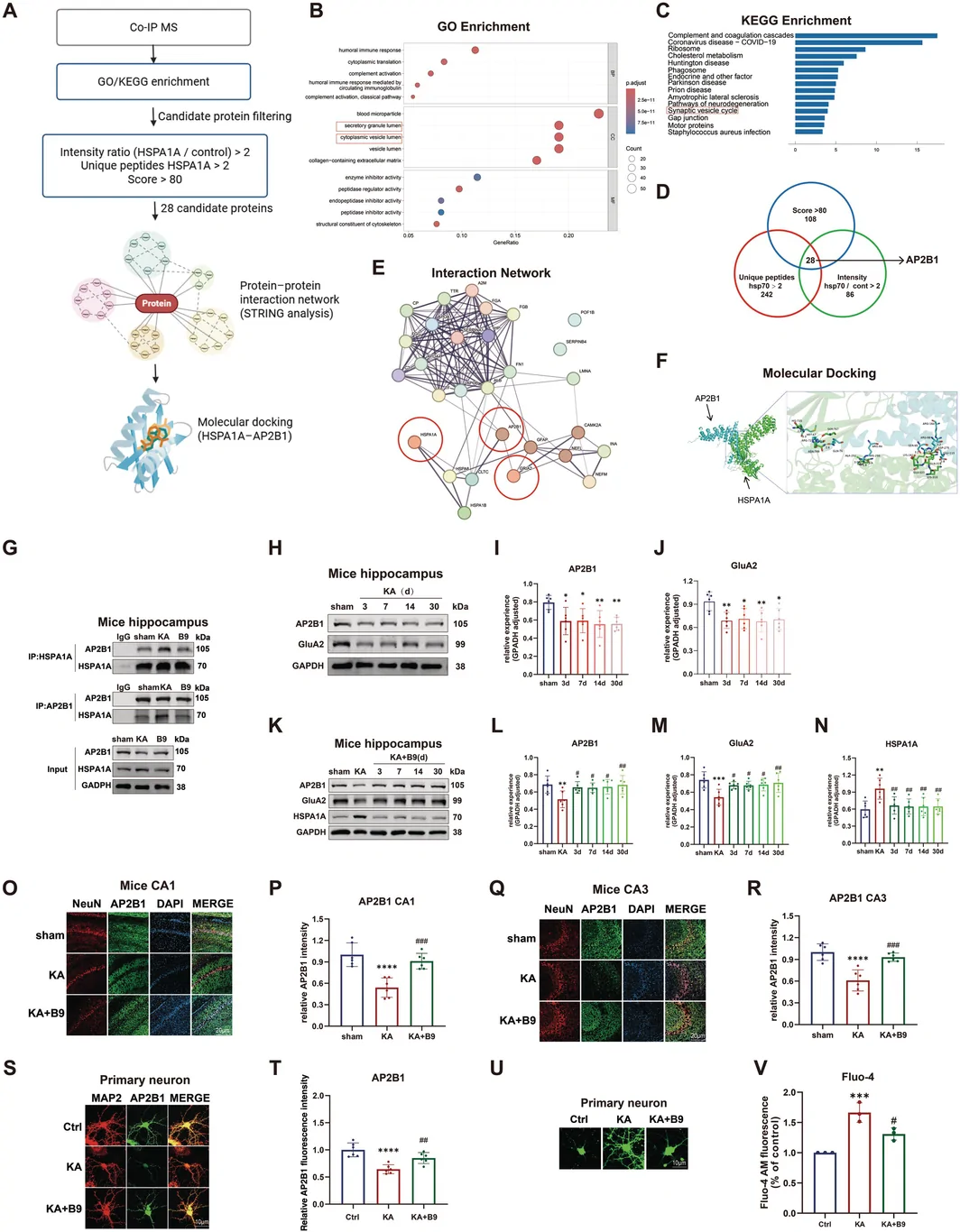
B9 disrupts the HSPA1A–AP2B1 interaction and alleviates epileptic Ca^2+^ overload. (A) Workflow for CoIP–MS screening, candidate filtering, protein–protein interaction analysis, and molecular docking. (B, C) GO and KEGG enrichment analyses of the filtered HSPA1A‐associated proteins. (D) Venn diagram of the candidate‐filtering criteria; AP2B1 is indicated. CoIP–MS was performed using sclerotic hippocampal samples from three independent patients with drug‐resistant TLE and was treated as a discovery analysis without between‐group inferential testing. (E) STRING protein–protein interaction network of the filtered candidates; HSPA1A, AP2B1, and GRIA2 are indicated. (F) Molecular docking model of HSPA1A and AP2B1. (G) Representative reciprocal co‐immunoprecipitation of HSPA1A and AP2B1 in mouse hippocampal lysates. IgG and input controls are shown. *n* = 6 mice per group; no quantitative comparison was performed. (H–J) Representative western blots and quantification of hippocampal AP2B1 and GluA2 at 3, 7, 14, and 30 days after KA injection. (K–N) Representative western blots and quantification of hippocampal AP2B1, GluA2, and HSPA1A in sham, KA, and KA + B9 mice at the indicated time points. *n* = 6 mice per group for panels H–N. (O, P) Representative NeuN/AP2B1 immunofluorescence images and quantification of AP2B1 fluorescence intensity in the hippocampal CA1 region. (Q, R) Representative NeuN/AP2B1 immunofluorescence images and quantification in the hippocampal CA3 region. *n* = 6 mice per group for panels O–R. Scale bars, 20 μm. (S, T) Representative MAP2/AP2B1 immunofluorescence images and quantification of AP2B1 fluorescence intensity in primary neurons. (U, V) Representative Fluo‐4 images and quantification of intracellular Ca^2+^ levels in primary neurons. For panels S‐T, *n* = 6 independent wells from a single primary‐neuron culture preparation per group. For panels U–V, *n* = 3 independent wells from a single primary‐neuron culture preparation per group. Scale bars, 10 μm. Quantitative data are presented as mean ± SEM. Panels I, J, and L–N were analyzed using one‐way ANOVA followed by Dunnett's multiple‐comparisons test. Panels P, R, T, and V were analyzed using one‐way ANOVA followed by Dunnett's multiple‐comparisons test. *p < 0.05, **p < 0.01, ***p < 0.001, and ****p < 0.0001 versus the corresponding sham or control group; ^#^ *p* < 0.05, ^##^ *p* < 0.01, and ^###^ *p* < 0.001 versus the corresponding KA group. Exact *p* values and the corresponding test statistics and degrees of freedom, where applicable, are provided in Table S3.

In addition, immunofluorescence staining showed spatial colocalization of HSPA1A and AP2B1 in the hippocampal CA1 and CA3 regions, as well as in primary neurons (Figure S2A–I). We next examined whether this interaction was altered under seizure‐associated conditions. Co‐immunoprecipitation assays qualitatively confirmed the association between HSPA1A and AP2B1 in mouse hippocampal tissues, with representative blots showing an apparently stronger association after KA treatment and a weaker association after B9 administration (Figure 3G). Consistently, western blot analysis showed that AP2B1 and GluA2 protein levels were significantly reduced in the hippocampus across the examined time points (Figure 3H–J). In contrast, B9 treatment significantly restored AP2B1 and GluA2 expression and significantly reduced HSPA1A levels in KA‐treated mice (Figure 3K–N). Immunofluorescence staining further demonstrated that AP2B1 expression was reduced in the CA1 and CA3 regions after KA treatment and significantly preserved by B9 (Figure 3O–R). A similar pattern was observed in primary cultured neurons, where KA significantly decreased AP2B1 immunoreactivity and B9 treatment significantly restored AP2B1 expression (Figure 3S,T). Because loss of GluA2 favors the formation of Ca^2+^‐permeable AMPARs, we next examined intracellular Ca^2+^ dynamics using Fluo‐4 imaging. KA stimulation induced prominent Ca^2+^ overload in primary neurons, whereas B9 significantly attenuated this increase (Figure 3U,V). Collectively, these findings indicate that HSPA1A interacts with AP2B1 and is associated with AP2B1/GluA2 disruption in the experimental models examined, whereas B9 interrupts the HSPA1A–AP2B1 interaction, preserves AP2B1 and GluA2 expression, and alleviates neuronal Ca^2+^ overload.

### 3.4 AP2B1 Knockdown Abolishes B9‐Mediated Attenuation of Seizure Susceptibility, Cognitive Impairment, and Neuronal Ca^2+^ Dysregulation

To determine whether AP2B1 is required for the observed functional and molecular effects of B9, we first established AP2B1 knockdown models using shRNA. The sh‐AP2B1 construct selected for subsequent experiments significantly reduced AP2B1 expression, confirming the knockdown efficiency (Figure 4A,B). In the hippocampus of sh‐AP2B1 mice, AP2B1 and GluA2 expression levels were significantly reduced, and B9 treatment did not significantly restore either protein (Figure 4C–E). Functionally, AP2B1 knockdown significantly increased seizure susceptibility in the cumulative PTZ challenge, and B9 did not significantly increase the number of PTZ injections required to reach the predefined Racine Stage IV/V endpoint in sh‐AP2B1 mice (Figure 4F,G). Consistently, in a separate acute PTZ/EEG cohort, the mean EEG envelope amplitude during the predefined 10‐min post‐PTZ analysis window was significantly increased in sh‐AP2B1 mice compared with B9‐treated mice, and additional B9 treatment did not significantly reduce this parameter after AP2B1 knockdown (Figure 4H,I). In the Barnes maze, AP2B1‐deficient mice showed significantly increased latency and navigation errors, whereas B9 did not significantly rescue either impairment; total locomotor distance did not differ significantly among the groups (Figure 4J–M). At the cellular level, AP2B1 knockdown significantly aggravated KA‐induced Ca^2+^ overload in primary neurons, and B9 did not significantly reduce Fluo‐4 fluorescence under this condition (Figure 4N,O). These results indicate that AP2B1 is required for the effects of B9 on evoked seizure susceptibility, cognitive performance, and neuronal Ca^2+^ homeostasis.

**FIGURE 4:**
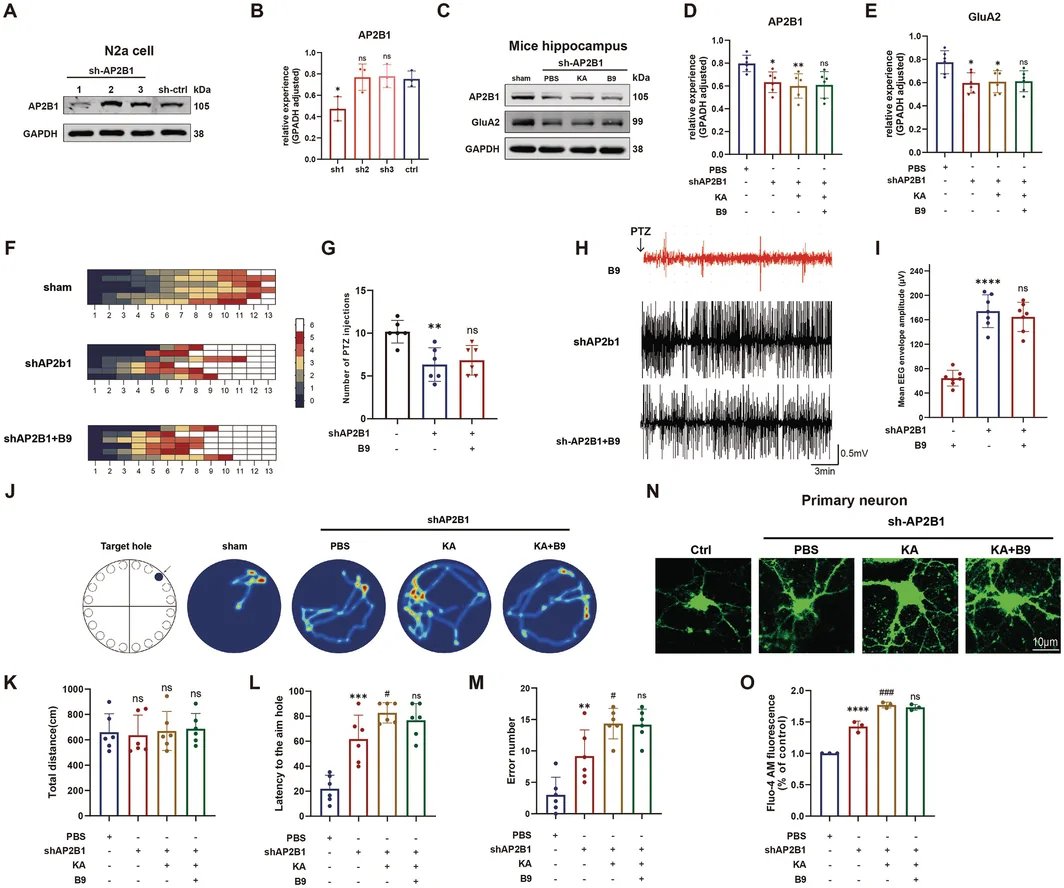
AP2B1 knockdown abolishes B9‐mediated attenuation of seizure susceptibility, cognitive impairment, and neuronal Ca^2+^ dysregulation. (A, B) Representative western blots and quantification of AP2B1 in N2a cells transfected with three sh‐AP2B1 constructs or control shRNA. *n* = 3 independent wells from a single N2a cell‐culture preparation per group. (C–E) Representative western blots and quantification of AP2B1 and GluA2 in hippocampal tissues from sham, sh‐AP2B1 + PBS, sh‐AP2B1 + KA, and sh‐AP2B1 + KA + B9 mice. (F, G) Heatmaps of Racine scores during the cumulative PTZ challenge and the number of PTZ injections required to reach the Racine Stage IV/V endpoint. (H, I) Representative post‐PTZ EEG traces and mean EEG envelope amplitude calculated over the predefined 10‐min analysis window for the B9, sh‐AP2B1, and sh‐AP2B1 + B9 groups. The values were derived from hippocampal EEG recordings, and a separate cohort was used for the acute PTZ/EEG experiment. (J) Representative Barnes maze trajectories. (K) Total distance traveled. (L) Latency to reach the target hole. (M) Number of navigation errors. *n* = 6 mice per group for panels C–M. (N, O) Representative Fluo‐4 images and quantification of intracellular Ca^2+^ levels in primary neurons with AP2B1 knockdown. *n* = 3 independent wells from a single primary‐neuron culture preparation per group. Scale bar, 10 μm. Quantitative data are presented as mean ± SEM. Panel B was analyzed using ordinary one‐way ANOVA followed by Dunnett's multiple‐comparisons test, with sh‐ctrl as the reference group. Panels G and I were analyzed using ordinary one‐way ANOVA followed by Dunnett's multiple‐comparisons test. Panels D, E, K–M, and O were analyzed using ordinary one‐way ANOVA followed by Šídák's multiple‐comparisons test for the prespecified comparisons. *p < 0.05, **p < 0.01, and ***p < 0.001 versus the corresponding sh‐ctrl, sham, or control group; ****p < 0.0001 versus the B9 group in panel I and versus the control group in panel O; #p < 0.05 and ###p < 0.001 versus the sh‐AP2B1 + PBS group; ns, not significant for the indicated comparisons. Exact *p* values and the corresponding test statistics and degrees of freedom, where applicable, are provided in Table S3.

### 3.5 AP2B1 Overexpression Reproduces the Functional and Molecular Effects of B9

To determine whether AP2B1 elevation is sufficient to mimic the action of B9, we next established an AAV‐mediated AP2B1 overexpression model in the hippocampus. Western blot analysis confirmed that AAV‐mediated AP2B1 overexpression significantly increased AP2B1 protein expression (Figure 5A,B). AP2B1 overexpression significantly increased AP2B1 and GluA2 expression under PBS‐treated conditions. Following KA treatment, AP2B1 and GluA2 levels did not differ significantly from the sham group, and additional B9 treatment produced no significant further change (Figure 5C–E). In the cumulative PTZ challenge, AP2B1 overexpression increased the number of PTZ injections required to reach the predefined Racine Stage IV/V endpoint, indicating reduced seizure susceptibility, and co‐treatment with B9 did not produce a significant further effect (Figure 5F,G). Similarly, in a separate acute PTZ/EEG cohort, AP2B1 overexpression significantly reduced the mean EEG envelope amplitude during the predefined 10‐min post‐PTZ analysis window, whereas additional B9 administration produced no significant further reduction (Figure 5H,I). No significant differences in total distance traveled, latency to the target hole, or number of navigation errors were detected among the examined groups in the Barnes maze (Figure 5J–M). In AP2B1‐overexpressing primary neurons, Fluo‐4 fluorescence did not differ significantly among the examined treatment conditions (Figure 5N,O). Together, these findings indicate that AP2B1 overexpression reproduces the effects of B9 on PTZ‐evoked seizure susceptibility and EEG activity, while additional B9 treatment produces no significant further effect under AP2B1‐overexpression conditions.

**FIGURE 5:**
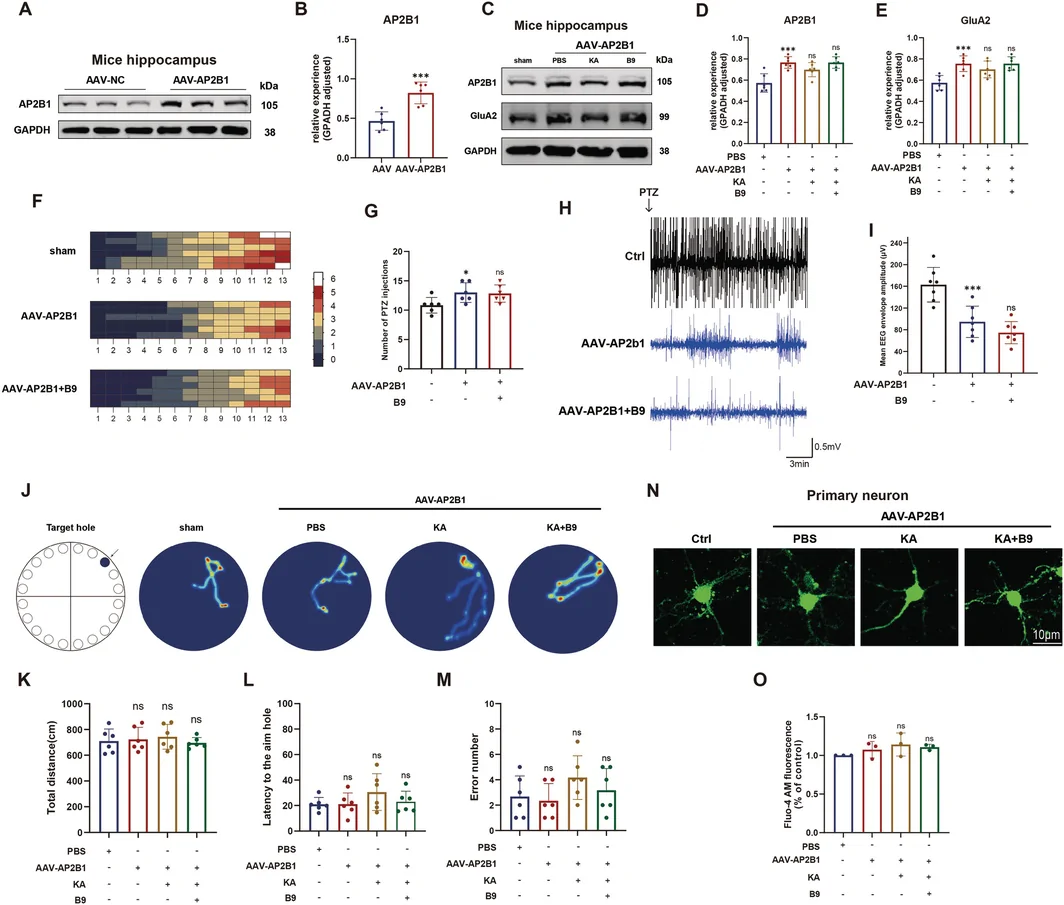
AP2B1 overexpression reproduces the functional and molecular effects of B9. (A, B) Representative western blots and quantification of AP2B1 in hippocampal tissues from AAV‐NC and AAV‐AP2B1 mice. *n* = 6 mice per group. (C–E) Representative western blots and quantification of AP2B1 and GluA2 in hippocampal tissues from sham, AAV‐AP2B1 + PBS, AAV‐AP2B1 + KA, and AAV‐AP2B1 + KA + B9 mice. (F, G) Heatmaps of Racine scores during the cumulative PTZ challenge and the number of PTZ injections required to reach the Racine Stage IV/V endpoint. (H, I) Representative post‐PTZ EEG traces and mean EEG envelope amplitude calculated over the predefined 10‐min analysis window and derived from hippocampal EEG recordings. A separate cohort was used for the acute PTZ/EEG experiment. (J) Representative Barnes maze trajectories. (K) Total distance traveled. (L) Latency to reach the target hole. (M) Number of navigation errors. *n* = 6 mice per group for panels C–M. (N, O) Representative Fluo‐4 images and quantification of intracellular Ca^2+^ levels in primary neurons with AP2B1 overexpression. *n* = 3 independent wells from a single primary‐neuron culture preparation per group. Scale bar, 10 μm. Quantitative data are presented as mean ± SEM. Panel B was analyzed using an unpaired two‐tailed Student's *t*‐test. Panels G and I were analyzed using ordinary one‐way ANOVA followed by Dunnett's multiple‐comparisons test. Panels D, E, K–M, and O were analyzed using ordinary one‐way ANOVA followed by Šídák's multiple‐comparisons test for the prespecified comparisons. *p < 0.05, **p < 0.01, and ***p < 0.001 versus the corresponding AAV‐NC, sham, or control group; ns, not significant for the indicated comparisons. Exact *p* values and the corresponding test statistics and degrees of freedom, where applicable, are provided in Table S3.

### 3.6 B9 Stabilizes AP2B1 Through O‐GlcNAcylation to Alleviate Neuronal Ca^2+^ Overload

To further explore how HSPA1A disrupts AP2B1, we first screened potential post‐translational modifications of AP2B1 using public databases. GeneCards analysis suggested that AP2B1 may undergo multiple post‐translational modifications, and GlyGen annotation further identified O‐GlcNAcylation as a candidate regulatory modification, with predicted sites at Ser671 and Ser672 (Figure 6A,B). Because O‐GlcNAcylation is known to regulate protein stability [22], we next examined whether AP2B1 might be regulated through this pathway under KA‐associated conditions. KA treatment significantly reduced hippocampal OGT and AP2B1 expression, whereas enhancement of O‐GlcNAcylation with Thiamet‐G [23, 24] significantly restored both proteins in KA‐treated mice (Figure 6C–E), indicating that O‐GlcNAcylation contributes to AP2B1 stability. In primary neurons, Thiamet‐G also significantly attenuated KA‐induced Ca^2+^ overload, as shown by reduced Fluo‐4 fluorescence (Figure 6F,G).

**FIGURE 6:**
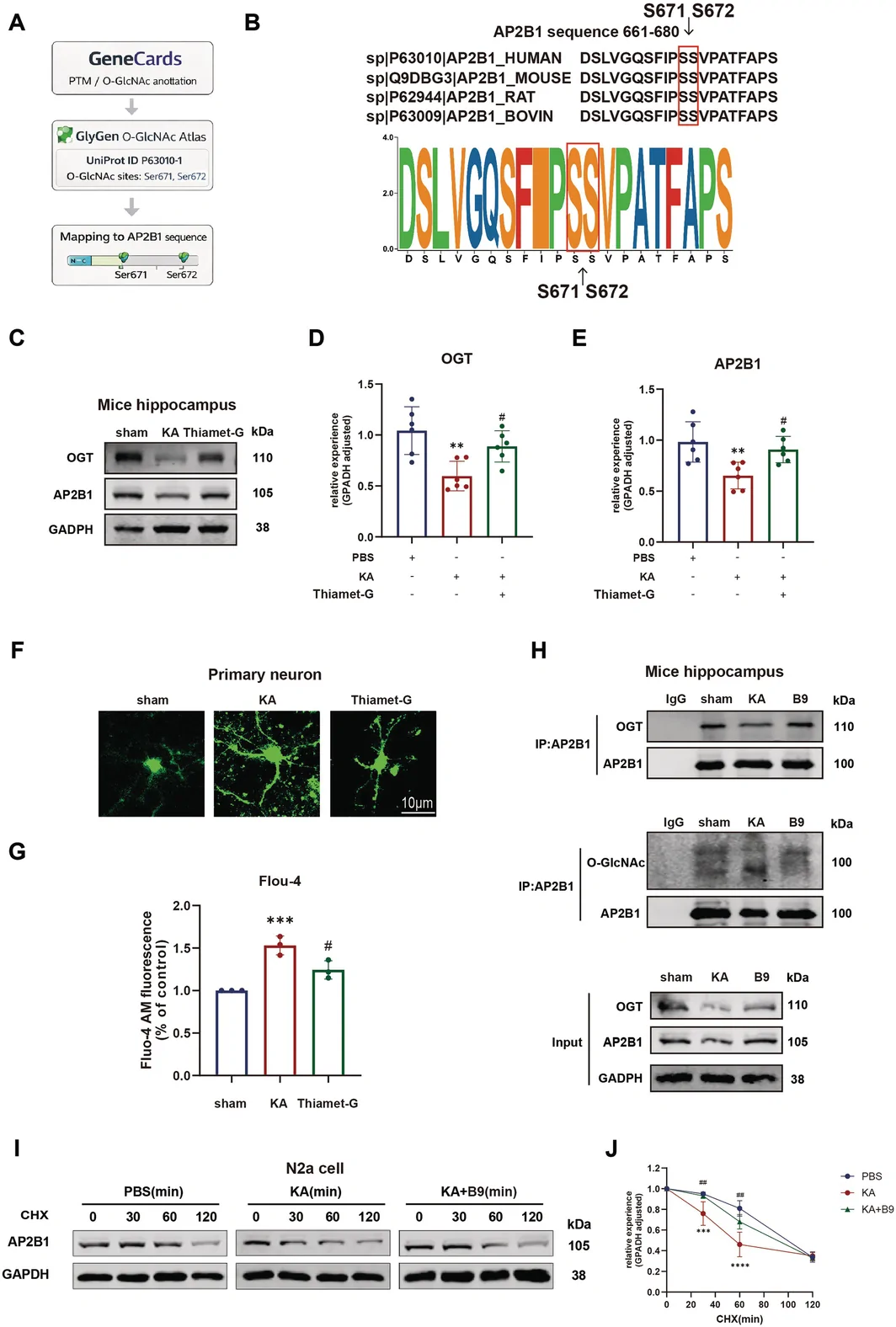
B9 stabilizes AP2B1 through O‐GlcNAcylation to alleviate neuronal Ca^2+^ overload. (A) Database‐based identification of candidate AP2B1 O‐GlcNAcylation sites using GeneCards and GlyGen. (B) Sequence alignment of AP2B1 residues 661–680 across species; the predicted candidate sites Ser671 and Ser672 are indicated. (C–E) Representative western blots and quantification of OGT and AP2B1 in hippocampal tissues from sham, KA, and Thiamet‐G‐treated mice. *n* = 6 mice per group. (F, G) Representative Fluo‐4 images and quantification of intracellular Ca^2+^ levels in primary neurons treated with KA and Thiamet‐G. *n* = 3 independent wells from a single primary‐neuron culture preparation per group. Scale bar, 10 μm. (H) Representative AP2B1 immunoprecipitation followed by immunoblotting for OGT, O‐GlcNAc, and AP2B1 in mouse hippocampal lysates. IgG and input controls are shown. *n* = 6 mice per group; no quantitative comparison was performed. (I, J) Representative cycloheximide chase assay and quantification of AP2B1 protein abundance in PBS‐, KA‐, and KA + B9‐treated N2a cells at the indicated time points. *n* = 6 independent wells from a single N2a cell‐culture preparation per group at each time point. Quantitative data are presented as mean ± SEM. Panels D, E, and G were analyzed using one‐way ANOVA followed by Dunnett's multiple‐comparisons test. Panel J was analyzed separately at 30, 60, and 120 min using ordinary one‐way ANOVA followed by Dunnett's multiple‐comparisons test, with KA as the reference group. **p < 0.01, ***p < 0.001 and ***p < 0.0001 versus the corresponding sham or PBS group; ^#^ *p* < 0.05 and ^##^ *p* < 0.01 versus the corresponding KA group. Exact *p* values and the corresponding test statistics and degrees of freedom, where applicable, are provided in Table S3.

To further assess whether B9 modulates AP2B1 O‐GlcNAcylation, we performed co‐immunoprecipitation assays in mouse hippocampal tissues. AP2B1 was found to associate with OGT, and KA reduced both the AP2B1–OGT interaction and AP2B1‐specific O‐GlcNAcylation. Representative co‐immunoprecipitation results qualitatively indicated that B9 restored AP2B1 association with OGT and increased AP2B1 O‐GlcNAcylation (Figure 6H), indicating that B9 promotes O‐GlcNAc‐dependent regulation of AP2B1. Finally, CHX chase assays showed that KA significantly accelerated AP2B1 degradation in N2a cells, whereas B9 significantly delayed this decline, indicating improved AP2B1 protein stability (Figure 6I,J). Together, these findings demonstrate that B9 stabilizes AP2B1 by restoring its O‐GlcNAcylation, thereby contributing to the alleviation of neuronal Ca^2+^ overload.

## 4 Discussion

Epileptogenesis is driven by progressive synaptic remodeling and maladaptive cellular stress responses that converge on neuronal hyperexcitability [25]. In the present study, systemic B9 treatment reduced PTZ‐evoked seizure susceptibility and ameliorated cognitive impairment in a separate KA‐treated cohort. These findings extend the investigation of B9—originally developed as an anti‐cancer agent—to a neurological disease context and identify the HSPA1A–AP2B1 axis as a stress‐signaling pathway associated with evoked seizure susceptibility and KA‐associated cognitive and molecular abnormalities. However, because spontaneous recurrent seizures and the chronic progression of epilepsy were not monitored, these findings should not be interpreted as evidence that B9 modifies the development or course of epilepsy.

Previous studies have described dual roles of HSPA1A in neurological disorders, where acute stress responses may exert protective effects, whereas sustained activation contributes to chronic pathology [26, 27, 28]. Consistent with this concept, persistent HSPA1A upregulation in our experimental models was accompanied by enhanced HSPA1A–AP2B1 association, reduced AP2B1 and GluA2 abundance, and neuronal Ca^2+^ overload. B9 was originally developed to suppress HSP70 transcription in tumor cells, particularly in glioblastoma, where it inhibits proliferation and survival [16, 17, 18]. In the present study, B9 reduced HSPA1A expression and was associated with preserved AP2B1 and GluA2 abundance and attenuated neuronal Ca^2+^ overload. The effects of HSPA1A modulation may therefore depend on the pathological context.

Adaptor protein complex 2 subunit beta 1 (AP2B1) is a component of the AP2 clathrin‐mediated endocytic complex and participates in synaptic receptor trafficking. AP2B1 has been implicated in synaptic dysfunction in Alzheimer's disease and other neuropsychiatric disorders [29, 30, 31]. Previous work demonstrated that AP2B1 knockdown reduced cellular GluA2 levels in developing rat hippocampal neurons through impaired mechanistic target of rapamycin (mTOR)‐dependent GluA2 biosynthesis, although CP‐AMPAR currents were not directly examined in that study [32]. Consistent with this previous observation, AP2B1 knockdown in the present study reduced GluA2 expression, whereas AP2B1 overexpression preserved GluA2 expression after KA treatment. However, the mechanisms governing AP2B1 protein stability under pathological neuronal excitation remain unclear. O‐GlcNAcylation regulates protein stability, stress adaptation, and neuronal signaling [23, 33, 34, 35]. Reduced O‐GlcNAcylation has been associated with Alzheimer's disease and Parkinson's disease [36, 37], but its role in epilepsy‐associated synaptic remodeling remains unclear.

Mechanistically, our data support a model in which sustained HSPA1A upregulation is associated with reduced AP2B1 O‐GlcNAcylation and stability. AP2B1 knockdown abolished several observed effects of B9, whereas AP2B1 overexpression reproduced the reductions in PTZ‐evoked seizure susceptibility and EEG activity observed with B9, supporting AP2B1 as an important downstream effector in the experimental paradigms examined. However, because CP‐AMPAR currents were not directly measured, the proposed link between GluA2 loss and CP‐AMPAR‐mediated Ca^2+^ influx remains inferential. B9 treatment enhanced AP2B1–OGT binding and AP2B1 O‐GlcNAcylation and was associated with restored AP2B1 stability, preserved GluA2 abundance, and attenuated seizure‐associated Ca^2+^ dysregulation.

Approximately 30%–40% of patients with epilepsy remain refractory to current antiseizure medications [38]. Although B9 reduced evoked seizure susceptibility, it should presently be considered a mechanism‐directed candidate for further evaluation rather than an established disease‐modifying treatment.

The cumulative PTZ challenge provided a controlled measure of evoked seizure susceptibility by comparing the PTZ exposure required to reach a predefined convulsive endpoint [39, 40]. Because PTZ induces seizures primarily by inhibiting GABAA receptor‐mediated neurotransmission, this paradigm does not measure spontaneous seizure generation [39]. In the KA‐treated cohorts, we assessed post‐KA cognitive and molecular abnormalities but did not electrographically characterize the acute KA‐induced seizure episode as status epilepticus or quantify the subsequent incidence and frequency of spontaneous recurrent seizures. Long‐term recordings have shown that characteristics of the initial status epilepticus are associated with the latency to spontaneous recurrent seizures in rats, whereas spontaneous seizures can emerge within 1–5 days after severe KA‐induced status epilepticus in C57BL/6J mice [41, 42]. However, these findings were obtained using systemic KA models and cannot be assumed to apply directly to the intracerebroventricular model used here. Accordingly, the post‐KA endpoints in the present study represent predefined time points for cognitive and molecular analyses rather than verified stages of epileptogenesis. Thus, the PTZ findings support an antiseizure effect on evoked seizure susceptibility but do not establish anti‐epileptogenic, disease‐modifying, or neuroprotective effects because spontaneous recurrent seizures and neuronal survival or loss were not quantified. Future studies using continuous video‐EEG from the acute KA insult through the chronic phase will be required to characterize the initial status epilepticus and determine whether post‐KA B9 treatment alters the latency, incidence, frequency, and duration of spontaneous recurrent seizures.

Longitudinal monitoring of spontaneous recurrent seizures has been used to evaluate putative anti‐epileptogenic and disease‐modifying treatments in post‐status epilepticus models of temporal lobe epilepsy [43]. In particular, Costa et al. reported that trilostane administered once daily for six consecutive days beginning shortly after KA‐induced status epilepticus delayed the onset of both electrographic and convulsive spontaneous recurrent seizures during long‐term video‐electrocorticographic (video‐ECoG) monitoring [44]. The delayed seizure onset occurred beyond the treatment period, whereas trilostane did not alter the initial status epilepticus or hippocampal neuronal loss, distinguishing its reported anti‐epileptogenic effect from acute antiseizure or neuroprotective effects [44]. In chronically epileptic rats with established spontaneous recurrent seizures, a subsequent study further suggested that a seven‐day course of trilostane attenuated the progression of spontaneous seizure occurrence by enhancing neurosteroid availability [45]. Trilostane increases endogenous neurosteroid availability, whereas B9 was examined as a modulator of HSPA1A–AP2B1–GluA2 signaling and neuronal Ca^2+^ homeostasis. Because the present study did not monitor spontaneous recurrent seizures, its findings cannot be directly compared with the reported anti‐epileptogenic or disease‐modifying effects of trilostane.

Several limitations warrant consideration. First, CP‐AMPAR involvement was inferred from changes in GluA2 abundance and intracellular Ca^2+^ levels rather than directly demonstrated. Direct electrophysiological measurements and selective pharmacological blockade were not performed; therefore, the data support a causal role for AP2B1 in regulating GluA2 abundance and neuronal Ca^2+^ homeostasis downstream of B9, whereas CP‐AMPAR‐specific regulation remains inferential. Second, the specific AP2B1 O‐GlcNAcylation sites were not identified in the present study. Third, only male mice were included in the in vivo experiments, which limits the generalizability of the findings to females. Future studies should validate these findings in female mice. Fourth, the 50‐Hz EEG sampling rate prevented reliable assessment of gamma‐range and higher‐frequency activity. Characterizing the spectral effects of B9 will require recordings at higher sampling rates with appropriate filtering. The human tissue comparison was also limited by the use of injured temporal cortex rather than non‐epileptic hippocampus; differences in anatomical region and injury background may therefore have contributed to the observed HSPA1A difference.

In summary, B9 reduced PTZ‐evoked seizure susceptibility and ameliorated KA‐associated cognitive and molecular abnormalities while modulating HSPA1A–AP2B1 signaling and neuronal Ca^2+^ homeostasis. These findings provide a mechanistic rationale for further investigation of B9 in epilepsy models but do not demonstrate that B9 alters the development or chronic progression of epilepsy. Longitudinal video‐EEG monitoring of spontaneous recurrent seizures will be required before any anti‐epileptogenic or disease‐modifying activity can be established.

## Author Contributions

**Zixiao Tan:** conceptualization, experimental design, data analysis, figure preparation, and manuscript drafting. **Qilong Zhai:** experimental investigation, animal model construction, molecular biology experiments, data acquisition, and data interpretation. **Jingwen Cui:** experimental investigation, assistance with animal surgery, behavioral experiments, and data collection. **Xiaoying Gao:** technical support for immunofluorescence experiments and data validation. **Xingan Liu:** assistance with electrophysiological data analysis and statistical evaluation. **Zhijie Fan:** support with viral vector construction and in vivo stereotactic injections. **Yu Liu:** provision of B9 peptide and related technical support, conceptual input, and critical revision of the manuscript. **Jiahang Sun:** conceptual supervision, funding acquisition, project administration, and critical revision of the manuscript. All authors read and approved the final version of the manuscript.

## Funding

This work was supported by the National Natural Science Foundation of China (Grant No. 81871016) and the Heilongjiang Provincial Natural Science Foundation of China (Joint Fund Cultivation Project, Grant No. PL2024H103).

## Ethics Statement

All animal experiments were approved by the Animal Ethics Committee of Harbin Medical University (approval no. 2024GZRYS‐294) and conducted in accordance with the National Institutes of Health Guide for the Care and Use of Laboratory Animals. The collection and use of human sclerotic hippocampal and injured temporal cortical tissues were approved by the Ethics Committee of the Second Affiliated Hospital of Harbin Medical University (approval no. KY2024‐125). All procedures involving human samples were conducted in accordance with the Declaration of Helsinki. Written informed consent was obtained from all participants or their legal guardians before sample collection, and all tissue samples and associated clinical data were de‐identified before analysis.

## Consent

Written informed consent was obtained from all participants or their legal guardians prior to sample collection.

## Conflicts of Interest

The authors declare no conflicts of interest.

## Supporting information


**Figure S1:** Seizure incidence in an orthotopic glioma model treated with B9.
**Figure S2:** HSPA1A and AP2B1 immunofluorescence in hippocampal regions and primary neurons.
**Table S1:** Modified Racine scale used for behavioral seizure scoring.
**Table S2:** Demographic and clinical characteristics of participants providing human brain tissue.
**Table S3:** Detailed statistical results for the quantitative analyses.

## Data Availability

The data that support the findings of this study are available from the corresponding author upon reasonable request.
