# Supplementary material for: Apoptin‐Derived Peptide Alleviates Temporal Lobe Epilepsy (TLE) by Limiting Neuronal Ca2+ Overload Through the HSPA1A–AP2B1 Axis
Source: CNS Neurosci Ther. 2026 Oct 3;32(10):e71193. doi: 10.1002/cns.71193 (PMC13633559; doi:10.1002/cns.71193)
Supplement: Supplementary file 1 [file CNS-32-e71193-s001.docx]

**Supplementary Material**

**Supplementary Figure S1. Seizure incidence in an orthotopic glioma model treated with B9.**

(A) Percentage of mice exhibiting seizures in vehicle- and B9-treated cohorts. Six independent cohorts were included per condition, with 10 mice in each cohort; each point represents one cohort. Data are presented as mean ± SEM. Seizure incidence at the cohort level was compared using an unpaired two-tailed Student’s t-test. **P < 0.01 versus the vehicle group. Exact P values and the corresponding test statistics and degrees of freedom, where applicable, are provided in Supplementary Table S3.

**Supplementary Figure S2. HSPA1A and AP2B1 immunofluorescence in hippocampal regions and primary neurons.**

(A–C) Representative HSPA1A/AP2B1 immunofluorescence images and quantification of HSPA1A and AP2B1 fluorescence intensity in the hippocampal CA1 region. (D–F) Representative HSPA1A/AP2B1 immunofluorescence images and quantification in the hippocampal CA3 region. n = 6 mice per group for panels A–F. Scale bars, 20 μm. (G–I) Representative HSPA1A/AP2B1 immunofluorescence images and quantification of HSPA1A and AP2B1 fluorescence intensity in primary neurons. n = 3 independent wells from a single primary-neuron culture preparation per group. Scale bar, 10 μm.

Quantitative data are presented as mean ± SEM and were analyzed using one-way ANOVA followed by Dunnett’s multiple-comparisons test. *P < 0.05, **P < 0.01, and ***P < 0.001 versus the corresponding sham or control group; #P < 0.05, ##P < 0.01, and ###P < 0.001 versus the corresponding KA group. Exact P values and the corresponding test statistics and degrees of freedom, where applicable, are provided in Supplementary Table S3.

**Supplementary Table S1. Modified Racine scale used for behavioral seizure scoring**

| Stage | Behavioral manifestation |
| --- | --- |
| 0 | No observable seizure behavior |
| I | Mouth and facial twitching, including rhythmic chewing, blinking, and whisker movements |
| II | Head nodding and/or facial clonus |
| III | Unilateral forelimb clonus without rearing |
| IV | Rearing accompanied by bilateral forelimb clonus |
| V | Rearing and falling with loss of postural control |
| VI | Generalized tonic–clonic seizure, potentially accompanied by wild running, jumping, or tonic extension |

Behavioral seizure severity was classified using a modified Racine scale with stages 0–VI.(1) The highest seizure stage observed during each assessment interval was recorded. During the cumulative PTZ challenge, the number of PTZ injections required to first reach stage IV or V was used as the predefined endpoint.

**Supplementary Table S2. Demographic and clinical characteristics of participants providing human brain tissue**

| **Patient code** | **Experimental use** | **Sex** | **Age at surgery (years)** | **Clinical diagnosis** | **Tissue analyzed** | **Histopathology** |
| --- | --- | --- | --- | --- | --- | --- |
| TLE-WB01 | WB, Fig. 2B–C | M | 52 | Drug-resistant TLE | Sclerotic hippocampus | Hippocampal sclerosis |
| TLE-WB02 | WB, Fig. 2B–C | M | 46 | Drug-resistant TLE | Sclerotic hippocampus | Hippocampal sclerosis |
| TLE-WB03 | WB, Fig. 2B–C | M | 37 | Drug-resistant TLE | Sclerotic hippocampus | Hippocampal sclerosis |
| TLE-WB04 | WB, Fig. 2B–C | F | 48 | Drug-resistant TLE | Sclerotic hippocampus | Hippocampal sclerosis |
| TLE-WB05 | WB, Fig. 2B–C | F | 55 | Drug-resistant TLE | Sclerotic hippocampus | Hippocampal sclerosis |
| TLE-WB06 | WB, Fig. 2B–C | F | 42 | Drug-resistant TLE | Sclerotic hippocampus | Hippocampal sclerosis |
| TBI-WB01 | WB, Fig. 2B–C | M | 58 | Traumatic brain injury without a history of epilepsy | Injured temporal cortex | Traumatic cortical injury |
| TBI-WB02 | WB, Fig. 2B–C | M | 64 | Traumatic brain injury without a history of epilepsy | Injured temporal cortex | Traumatic cortical injury |
| TBI-WB03 | WB, Fig. 2B–C | M | 57 | Traumatic brain injury without a history of epilepsy | Injured temporal cortex | Traumatic cortical injury |
| TBI-WB04 | WB, Fig. 2B–C | F | 66 | Traumatic brain injury without a history of epilepsy | Injured temporal cortex | Traumatic cortical injury |
| TBI-WB05 | WB, Fig. 2B–C | F | 62 | Traumatic brain injury without a history of epilepsy | Injured temporal cortex | Traumatic cortical injury |
| TBI-WB06 | WB, Fig. 2B–C | F | 52 | Traumatic brain injury without a history of epilepsy | Injured temporal cortex | Traumatic cortical injury |
| TLE-MS01 | CoIP–MS | M | 53 | Drug-resistant TLE | Sclerotic hippocampus | Hippocampal sclerosis |
| TLE-MS02 | CoIP–MS | M | 48 | Drug-resistant TLE | Sclerotic hippocampus | Hippocampal sclerosis |
| TLE-MS03 | CoIP–MS | F | 58 | Drug-resistant TLE | Sclerotic hippocampus | Hippocampal sclerosis |

**Supplementary Table S3. Detailed statistical results for the quantitative analyses.**

| **Figure/panel** | **comparison** | **Statistical test** | **Test statistic** | **df** | **P value** |
| --- | --- | --- | --- | --- | --- |
| Figure 1C | B9 vs PBS | Unpaired two-tailed Student’s t-test | t = 4.143 | 10 | 0.0020 |
| Figure 1E | B9 vs PBS | Unpaired two-tailed Student’s t-test | t = 4.404 | 10 | 0.0013 |
| Figure 1H | Overall group effect | One-way ANOVA | F = 0.3310 | 2, 15 | 0.7233 |
| Figure 1H | sham vs KA | Dunnett’s multiple-comparisons test | q = 0.3427 | 15 | 0.9185 (adjusted) |
| Figure 1H | KA + B9 vs KA | Dunnett’s multiple-comparisons test | q = 0.4677 | 15 | 0.8553 (adjusted) |
| Figure 1I | Overall group effect | Welch’s one-way ANOVA | W = 13.61 | 2, 8.959 | 0.0019 |
| Figure 1I | KA vs sham | Dunnett’s T3 multiple-comparisons test | t = 5.026 | 6.087 | 0.0046 (adjusted) |
| Figure 1I | KA vs KA + B9 | Dunnett’s T3 multiple-comparisons test | t = 3.291 | 7.121 | 0.0253 (adjusted) |
| Figure 1J | Overall group effect | One-way ANOVA | F = 12.54 | 2, 15 | 0.0006 |
| Figure 1J | KA vs sham | Dunnett’s multiple-comparisons test | q = 4.911 | 15 | 0.0004 (adjusted) |
| Figure 1J | KA vs KA + B9 | Dunnett’s multiple-comparisons test | q = 3.305 | 15 | 0.0090 (adjusted) |
| Figure 2C | TLE vs TBI | Unpaired two-tailed Student’s t-test | t = 2.429 | 10 | 0.0355 |
| Figure 2E | Overall group effect | Dunnett’s multiple-comparisons test | F = 8.853 | 4, 25 | 0.0001 |
| Figure 2E | sham vs 3d | Dunnett’s multiple-comparisons test | q = 5.015 | 25 | 0.0001 (adjusted) |
| Figure 2E | sham vs 7d | Dunnett’s multiple-comparisons test | q = 4.367 | 25 | 0.0007 (adjusted) |
| Figure 2E | sham vs 14d | Dunnett’s multiple-comparisons test | q = 4.141 | 25 | 0.0013 (adjusted) |
| Figure 2E | sham vs 30d | Dunnett’s multiple-comparisons test | q = 4.983 | 25 | 0.0001 (adjusted) |
| Figure 2G | Overall group effect | One-way ANOVA | F = 4.938 | 5, 30 | 0.0020 |
| Figure 2G | KA vs sham | Dunnett’s multiple-comparisons test | q = 3.988 | 30 | 0.0018 (adjusted) |
| Figure 2G | KA vs KA + B9 (3 d) | Dunnett’s multiple-comparisons test | q = 3.939 | 30 | 0.0020 (adjusted) |
| Figure 2G | KA vs KA + B9 (7 d) | Dunnett’s multiple-comparisons test | q = 4.056 | 30 | 0.0015 (adjusted) |
| Figure 2G | KA vs KA + B9 (14 d) | Dunnett’s multiple-comparisons test | q = 3.671 | 30 | 0.0042 (adjusted) |
| Figure 2G | KA vs KA + B9 (30 d) | Dunnett’s multiple-comparisons test | q = 3.040 | 30 | 0.0205 (adjusted) |
| Figure 2I | Overall group effect | Welch’s one-way ANOVA | W = 6.020 | 2, 8.405 | 0.0239 |
| Figure 2I | KA vs sham | Dunnett’s T3 multiple-comparisons test | t = 3.428 | 9.869 | 0.0126 (adjusted) |
| Figure 2I | KA vs KA + B9 | Dunnett’s T3 multiple-comparisons test | t = 2.974 | 6.853 | 0.0392 (adjusted) |
| Figure 2K | Overall group effect | One-way ANOVA | F = 13.33 | 2, 15 | 0.0005 |
| Figure 2K | KA vs sham | Dunnett’s multiple-comparisons test | q = 4.973 | 15 | 0.0003 (adjusted) |
| Figure 2K | KA vs KA + B9 | Dunnett’s multiple-comparisons test | q = 3.689 | 15 | 0.0041 (adjusted) |
| Figure 2M | Overall group effect | One-way ANOVA | F = 16.28 | 2, 15 | 0.0002 |
| Figure 2M | KA vs control | Dunnett’s multiple-comparisons test | q = 5.583 | 15 | 0.0001 (adjusted) |
| Figure 2M | KA vs KA + B9 | Dunnett’s multiple-comparisons test | q = 3.815 | 15 | 0.0032 (adjusted) |
| Figure 3I | Overall group effect | One-way ANOVA | F = 4.056 | 4, 25 | 0.0114 |
| Figure 3I | sham vs 3 d | Dunnett’s multiple-comparisons test | q = 2.923 | 25 | 0.0247 (adjusted) |
| Figure 3I | sham vs 7 d | Dunnett’s multiple-comparisons test | q = 2.870 | 25 | 0.0279 (adjusted) |
| Figure 3I | sham vs 14 d | Dunnett’s multiple-comparisons test | q = 3.393 | 25 | 0.0082 (adjusted) |
| Figure 3I | sham vs 30 d | Dunnett’s multiple-comparisons test | q = 3.365 | 25 | 0.0087 (adjusted) |
| Figure 3J | Overall group effect | One-way ANOVA | F = 4.339 | 4, 25 | 0.0084 |
| Figure 3J | sham vs 3 d | Dunnett’s multiple-comparisons test | q = 3.371 | 25 | 0.0086 (adjusted) |
| Figure 3J | sham vs 7 d | Dunnett’s multiple-comparisons test | q = 3.061 | 25 | 0.0180 (adjusted) |
| Figure 3J | sham vs 14 d | Dunnett’s multiple-comparisons test | q = 3.505 | 25 | 0.0062 (adjusted) |
| Figure 3J | sham vs 30 d | Dunnett’s multiple-comparisons test | q = 3.141 | 25 | 0.0149 (adjusted) |
| Fig. 3L | Overall comparison | Ordinary one-way ANOVA | F = 3.205 | 5, 30 | 0.0195 |
| Fig. 3L | KA vs sham | Dunnett’s multiple-comparisons test | q = 3.400 | 30 | 0.0084 (adjusted) |
| Fig. 3L | KA vs KA + B9 (3 d) | Dunnett’s multiple-comparisons test | q = 2.762 | 30 | 0.0394 (adjusted) |
| Fig. 3L | KA vs KA + B9 (7 d) | Dunnett’s multiple-comparisons test | q = 2.673 | 30 | 0.0483 (adjusted) |
| Fig. 3L | KA vs KA + B9 (14 d) | Dunnett’s multiple-comparisons test | q = 2.880 | 30 | 0.0300 (adjusted) |
| Fig. 3L | KA vs KA + B9 (30 d) | Dunnett’s multiple-comparisons test | q = 3.342 | 30 | 0.0097 (adjusted) |
| Fig. 3M | Overall comparison | Ordinary one-way ANOVA | F = 4.301 | 5, 30 | 0.0046 |
| Fig. 3M | KA vs sham | Dunnett’s multiple-comparisons test | q = 4.278 | 30 | 0.0008 (adjusted) |
| Fig. 3M | KA vs KA + B9 (3 d) | Dunnett’s multiple-comparisons test | q = 2.959 | 30 | 0.0248 (adjusted) |
| Fig. 3M | KA vs KA + B9 (7 d) | Dunnett’s multiple-comparisons test | q = 2.916 | 30 | 0.0275 (adjusted) |
| Fig. 3M | KA vs KA + B9 (14 d) | Dunnett’s multiple-comparisons test | q = 3.121 | 30 | 0.0168 (adjusted) |
| Fig. 3M | KA vs KA + B9 (30 d) | Dunnett’s multiple-comparisons test | q = 3.565 | 30 | 0.0055 (adjusted) |
| Fig. 3N | Overall comparison | Ordinary one-way ANOVA | F = 4.512 | 5, 30 | 0.0035 |
| Fig. 3N | KA vs sham | Dunnett’s multiple-comparisons test | q = 4.113 | 30 | 0.0013 (adjusted) |
| Fig. 3N | KA vs KA + B9 (3 d) | Dunnett’s multiple-comparisons test | q = 3.362 | 30 | 0.0092 (adjusted) |
| Fig. 3N | KA vs KA + B9 (7 d) | Dunnett’s multiple-comparisons test | q = 3.556 | 30 | 0.0056 (adjusted) |
| Fig. 3N | KA vs KA + B9 (14 d) | Dunnett’s multiple-comparisons test | q = 3.554 | 30 | 0.0056 (adjusted) |
| Fig. 3N | KA vs KA + B9 (30 d) | Dunnett’s multiple-comparisons test | q = 3.544 | 30 | 0.0058 (adjusted) |
| Fig. 3P | Overall comparison | Ordinary one-way ANOVA | F = 18.55 | 2, 15 | <0.0001 |
| Fig. 3P | KA vs sham | Dunnett’s multiple-comparisons test | q = 5.746 | 15 | <0.0001 (adjusted) |
| Fig. 3P | KA vs KA + B9 | Dunnett’s multiple-comparisons test | q = 4.622 | 15 | 0.0006 (adjusted) |
| Fig. 3R | Overall comparison | Ordinary one-way ANOVA | F = 20.82 | 2, 15 | <0.0001 |
| Fig. 3R | KA vs sham | Dunnett’s multiple-comparisons test | q = 6.051 | 15 | <0.0001 (adjusted) |
| Fig. 3R | KA vs KA + B9 | Dunnett’s multiple-comparisons test | q = 4.965 | 15 | 0.0003 (adjusted) |
| Fig. 3T | Overall comparison | Ordinary one-way ANOVA | F = 17.49 | 2, 15 | 0.0001 |
| Fig. 3T | KA vs control | Dunnett’s multiple-comparisons test | q = 5.890 | 15 | <0.0001 (adjusted) |
| Fig. 3T | KA vs KA + B9 | Dunnett’s multiple-comparisons test | q = 3.413 | 15 | 0.0072 (adjusted) |
| Fig. 3V | Overall comparison | Ordinary one-way ANOVA | F = 27.25 | 2, 6 | 0.0010 |
| Fig. 3V | KA vs control | Dunnett’s multiple-comparisons test | q = 7.377 | 6 | 0.0006 (adjusted) |
| Fig. 3V | KA vs KA + B9 | Dunnett’s multiple-comparisons test | q = 3.935 | 6 | 0.0136 (adjusted) |
| Fig. 4B | Overall comparison | Ordinary one-way ANOVA | F = 5.736 | 3, 8 | 0.0216 |
| Fig. 4B | sh-ctrl vs sh1 | Dunnett’s multiple-comparisons test | q = 3.211 | 8 | 0.0307 (adjusted) |
| Fig. 4B | sh-ctrl vs sh2 | Dunnett’s multiple-comparisons test | q = 0.1950 | 8 | 0.9944 (adjusted) |
| Fig. 4B | sh-ctrl vs sh3 | Dunnett’s multiple-comparisons test | q = 0.3034 | 8 | 0.9800 (adjusted) |
| Fig. 4D | Overall comparison | Ordinary one-way ANOVA | F = 5.354 | 3, 20 | 0.0072 |
| Fig. 4D | PBS vs sh-AP2B1 | Šídák’s multiple-comparisons test | t = 2.899 | 20 | 0.0264 (adjusted) |
| Fig. 4D | PBS vs sh-AP2B1 + KA | Šídák’s multiple-comparisons test | t = 3.484 | 20 | 0.0070 (adjusted) |
| Fig. 4D | sh-AP2B1 + KA vs sh-AP2B1 + KA + B9 | Šídák’s multiple-comparisons test | t = 0.1629 | 20 | 0.9979 (adjusted) |
| Fig. 4E | Overall comparison | Ordinary one-way ANOVA | F = 5.012 | 3, 20 | 0.0094 |
| Fig. 4E | PBS vs sh-AP2B1 | Šídák’s multiple-comparisons test | t = 3.319 | 20 | 0.0102 (adjusted) |
| Fig. 4E | PBS vs sh-AP2B1 + KA | Šídák’s multiple-comparisons test | t = 3.114 | 20 | 0.0163 (adjusted) |
| Fig. 4E | sh-AP2B1 + KA vs sh-AP2B1 + KA + B9 | Šídák’s multiple-comparisons test | t = 0.07538 | 20 | 0.9998 (adjusted) |
| Fig. 4G | Overall comparison | Ordinary one-way ANOVA | F = 9.089 | 2, 15 | 0.0026 |
| Fig. 4G | sh-AP2B1 vs sham | Dunnett’s multiple-comparisons test | q = 3.921 | 15 | 0.0026 (adjusted) |
| Fig. 4G | sh-AP2B1 vs sh-AP2B1 + B9 | Dunnett’s multiple-comparisons test | q = 0.5115 | 15 | 0.8303 (adjusted) |
| Fig. 4I | Overall comparison | Ordinary one-way ANOVA | F = 52.97 | 2, 18 | <0.0001 |
| Fig. 4I | sh-AP2B1 vs B9 | Dunnett’s multiple-comparisons test | q = 9.289 | 18 | <0.0001 (adjusted) |
| Fig. 4I | sh-AP2B1 vs sh-AP2B1 + B9 | Dunnett’s multiple-comparisons test | q = 0.8043 | 18 | 0.6421 (adjusted) |
| Fig. 4K | Overall comparison | Ordinary one-way ANOVA | F = 0.1268 | 3, 20 | 0.9431 |
| Fig. 4K | PBS vs sh-AP2B1 | Šídák’s multiple-comparisons test | t = 0.2790 | 20 | 0.9898 (adjusted) |
| Fig. 4K | sh-AP2B1 vs sh-AP2B1 + KA | Šídák’s multiple-comparisons test | t = 0.3982 | 20 | 0.9715 (adjusted) |
| Fig. 4K | sh-AP2B1 + KA vs sh-AP2B1 + KA + B9 | Šídák’s multiple-comparisons test | t = 0.2047 | 20 | 0.9959 (adjusted) |
| Fig. 4L | Overall comparison | Ordinary one-way ANOVA | F = 24.70 | 3, 20 | <0.0001 |
| Fig. 4L | PBS vs sh-AP2B1 | Šídák’s multiple-comparisons test | t = 5.106 | 20 | 0.0002 (adjusted) |
| Fig. 4L | sh-AP2B1 vs sh-AP2B1 + KA | Šídák’s multiple-comparisons test | t = 2.703 | 20 | 0.0405 (adjusted) |
| Fig. 4L | sh-AP2B1 + KA vs sh-AP2B1 + KA + B9 | Šídák’s multiple-comparisons test | t = 0.7740 | 20 | 0.8318 (adjusted) |
| Fig. 4M | Overall comparison | Ordinary one-way ANOVA | F = 18.34 | 3, 20 | <0.0001 |
| Fig. 4M | PBS vs sh-AP2B1 | Šídák’s multiple-comparisons test | t = 3.493 | 20 | 0.0069 (adjusted) |
| Fig. 4M | sh-AP2B1 vs sh-AP2B1 + KA | Šídák’s multiple-comparisons test | t = 2.927 | 20 | 0.0248 (adjusted) |
| Fig. 4M | sh-AP2B1 + KA vs sh-AP2B1 + KA + B9 | Šídák’s multiple-comparisons test | t = 0.09441 | 20 | 0.9996 (adjusted) |
| Fig. 4O | Overall comparison | Ordinary one-way ANOVA | F = 129.7 | 3, 8 | <0.0001 |
| Fig. 4O | PBS vs sh-AP2B1 | Šídák’s multiple-comparisons test | t = 9.635 | 8 | <0.0001 (adjusted) |
| Fig. 4O | sh-AP2B1 vs sh-AP2B1 + KA | Šídák’s multiple-comparisons test | t = 7.753 | 8 | 0.0002 (adjusted) |
| Fig. 4O | sh-AP2B1 + KA vs sh-AP2B1 + KA + B9 | Šídák’s multiple-comparisons test | t = 0.8280 | 8 | 0.8164 (adjusted) |
| Fig. 5B | AAV-AP2B1 vs AAV-NC | Unpaired two-tailed Student’s t-test | t = 4.834 | 10 | 0.0007 |
| Fig. 5D | Overall comparison | Ordinary one-way ANOVA | F = 11.06 | 3, 20 | 0.0002 |
| Fig. 5D | sham vs AAV-AP2B1 | Šídák’s multiple-comparisons test | t = 4.995 | 20 | 0.0002 (adjusted) |
| Fig. 5D | AAV-AP2B1 vs AAV-AP2B1 + KA | Šídák’s multiple-comparisons test | t = 1.754 | 20 | 0.2580 (adjusted) |
| Fig. 5D | AAV-AP2B1 + KA vs AAV-AP2B1 + KA + B9 | Šídák’s multiple-comparisons test | t = 1.741 | 20 | 0.2636 (adjusted) |
| Fig. 5E | Overall comparison | Ordinary one-way ANOVA | F = 8.454 | 3, 20 | 0.0008 |
| Fig. 5E | sham vs AAV-AP2B1 | Šídák’s multiple-comparisons test | t = 4.347 | 20 | 0.0009 (adjusted) |
| Fig. 5E | AAV-AP2B1 vs AAV-AP2B1 + KA | Šídák’s multiple-comparisons test | t = 1.299 | 20 | 0.5047 (adjusted) |
| Fig. 5E | AAV-AP2B1 + KA vs AAV-AP2B1 + KA + B9 | Šídák’s multiple-comparisons test | t = 1.323 | 20 | 0.4894 (adjusted) |
| Figure 5G | Overall comparison among three groups | Ordinary one-way ANOVA | F = 3.886 | 2, 15 | 0.0437 |
| Figure 5G | AAV-AP2B1 vs sham | Dunnett’s multiple comparisons test | q = 2.505 | 15 | 0.0441 (adjusted) |
| Figure 5G | AAV-AP2B1 vs AAV-AP2B1 + B9 | Dunnett’s multiple comparisons test | q = 0.1927 | 15 | 0.9732 (adjusted) |
| Figure 5I | Overall comparison among three groups | Ordinary one-way ANOVA | F = 19.85 | 2, 18 | <0.0001 (exact value required) |
| Figure 5I | AAV-AP2B1 vs Ctrl | Dunnett’s multiple comparisons test | q = 4.649 | 18 | 0.0004 (adjusted) |
| Figure 5I | AAV-AP2B1 vs AAV-AP2B1 + B9 | Dunnett’s multiple comparisons test | q = 1.358 | 18 | 0.3167 (adjusted) |
| Figure 5K | Overall comparison among four groups | Ordinary one-way ANOVA | F = 0.3327 | 3, 20 | 0.8018 |
| Figure 5K | sham vs AAV-AP2B1 | Šídák’s multiple comparisons test | t = 0.2743 | 20 | 0.9903 (adjusted) |
| Figure 5K | AAV-AP2B1 vs AAV-AP2B1 + KA | Šídák’s multiple comparisons test | t = 0.3934 | 20 | 0.9725 (adjusted) |
| Figure 5K | AAV-AP2B1 + KA vs AAV-AP2B1 + KA + B9 | Šídák’s multiple comparisons test | t = 0.9578 | 20 | 0.7249 (adjusted) |
| Figure 5L | Overall comparison among four groups | Ordinary one-way ANOVA | F = 1.285 | 3, 20 | 0.3066 |
| Figure 5L | sham vs AAV-AP2B1 | Šídák’s multiple comparisons test | t = 0.02764 | 20 | >0.9999 (adjusted; exact value required) |
| Figure 5L | AAV-AP2B1 vs AAV-AP2B1 + KA | Šídák’s multiple comparisons test | t = 1.670 | 20 | 0.2964 (adjusted) |
| Figure 5L | AAV-AP2B1 + KA vs AAV-AP2B1 + KA + B9 | Šídák’s multiple comparisons test | t = 1.349 | 20 | 0.4733 (adjusted) |
| Figure 5M | Overall comparison among four groups | Ordinary one-way ANOVA | F = 1.465 | 3, 20 | 0.2542 |
| Figure 5M | sham vs AAV-AP2B1 | Šídák’s multiple comparisons test | t = 0.3569 | 20 | 0.9792 (adjusted) |
| Figure 5M | AAV-AP2B1 vs AAV-AP2B1 + KA | Šídák’s multiple comparisons test | t = 1.963 | 20 | 0.1792 (adjusted) |
| Figure 5M | AAV-AP2B1 + KA vs AAV-AP2B1 + KA + B9 | Šídák’s multiple comparisons test | t = 1.071 | 20 | 0.6526 (adjusted) |
| Figure 5O | Overall comparison among four groups | Ordinary one-way ANOVA | F = 1.240 | 3, 8 | 0.3575 |
| Figure 5O | Ctrl vs AAV-AP2B1 | Šídák’s multiple comparisons test | t = 0.9648 | 8 | 0.7414 (adjusted) |
| Figure 5O | AAV-AP2B1 vs AAV-AP2B1 + KA | Šídák’s multiple comparisons test | t = 0.8774 | 8 | 0.7902 (adjusted) |
| Figure 5O | AAV-AP2B1 + KA vs AAV-AP2B1 + KA + B9 | Šídák’s multiple comparisons test | t = 0.4420 | 8 | 0.9641 (adjusted) |
| Figure 6D | Overall comparison among three groups | Ordinary one-way ANOVA | F = 9.288 | 2, 15 | 0.0024 |
| Figure 6D | KA vs sham | Dunnett’s multiple comparisons test | q = 4.245 | 15 | 0.0013 (adjusted) |
| Figure 6D | KA vs Thiamet-G | Dunnett’s multiple comparisons test | q = 2.768 | 15 | 0.0264 (adjusted) |
| Figure 6E | Overall comparison among three groups | Ordinary one-way ANOVA | F = 7.386 | 2, 15 | 0.0059 |
| Figure 6E | KA vs sham | Dunnett’s multiple comparisons test | q = 3.664 | 15 | 0.0043 (adjusted) |
| Figure 6E | KA vs KA + Thiamet-G | Dunnett’s multiple comparisons test | q = 2.836 | 15 | 0.0231 (adjusted) |
| Figure 6G | Overall comparison among three groups | Ordinary one-way ANOVA | F = 26.77 | 2, 6 | 0.0010 |
| Figure 6G | KA vs Ctrl | Dunnett’s multiple comparisons test | q = 7.308 | 6 | 0.0006 (adjusted) |
| Figure 6G | KA vs KA + Thiamet-G | Dunnett’s multiple comparisons test | q = 3.959 | 6 | 0.0133 (adjusted) |
| Figure 6J (30 min) | Overall comparison among three groups | Ordinary one-way ANOVA | F = 13.91 | 2, 15 | 0.0004 |
| Figure 6J (30 min) | KA vs PBS | Dunnett’s multiple comparisons test | q = 4.799 | 15 | 0.0004 (adjusted) |
| Figure 6J (30 min) | KA vs KA + B9 | Dunnett’s multiple comparisons test | q = 4.294 | 15 | 0.0012 (adjusted) |
| Figure 6J (60 min) | Overall comparison among three groups | Ordinary one-way ANOVA | F = 22.77 | 2, 15 | <0.0001 |
| Figure 6J (60 min) | KA vs PBS | Dunnett’s multiple comparisons test | q = 6.675 | 15 | <0.0001 (adjusted) |
| Figure 6J (60 min) | KA vs KA + B9 | Dunnett’s multiple comparisons test | q = 4.196 | 15 | 0.0015 (adjusted) |
| Figure 6J (120 min) | Overall comparison among three groups | Ordinary one-way ANOVA | F = 0.4058 | 2, 15 | 0.6736 |
| Figure 6J (120 min) | KA vs PBS | Dunnett’s multiple comparisons test | q = 0.5897 | 15 | 0.7829 (adjusted) |
| Figure 6J (120 min) | KA vs KA + B9 | Dunnett’s multiple comparisons test | q = 0.8846 | 15 | 0.5909 (adjusted) |
| Figure S1A | B9 vs Vehicle | Unpaired two-tailed Student’s t-test | t = 4.243 | 10 | 0.0017 |
| Figure S2B | Overall comparison among three groups | Ordinary one-way ANOVA | F = 22.82 | 2, 15 | <0.0001 |
| Figure S2B | KA vs sham | Dunnett’s multiple comparisons test | q = 6.727 | 15 | <0.0001 (adjusted) |
| Figure S2B | KA vs KA + B9 | Dunnett’s multiple comparisons test | q = 3.914 | 15 | 0.0026 (adjusted) |
| Figure S2C | Overall comparison among three groups | Ordinary one-way ANOVA | F = 7.018 | 2, 15 | 0.0071 |
| Figure S2C | KA vs sham | Dunnett’s multiple comparisons test | q = 3.643 | 15 | 0.0045 (adjusted) |
| Figure S2C | KA vs KA + B9 | Dunnett’s multiple comparisons test | q = 2.580 | 15 | 0.0381 (adjusted) |
| Figure S2E | Overall comparison among three groups | Ordinary one-way ANOVA | F = 13.60 | 2, 15 | 0.0004 |
| Figure S2E | KA vs sham | Dunnett’s multiple comparisons test | q = 5.182 | 15 | 0.0002 (adjusted) |
| Figure S2E | KA vs KA + B9 | Dunnett’s multiple comparisons test | q = 3.105 | 15 | 0.0135 (adjusted) |
| Figure S2F | Overall comparison among three groups | Ordinary one-way ANOVA | F = 9.625 | 2, 15 | 0.0020 |
| Figure S2F | KA vs sham | Dunnett’s multiple comparisons test | q = 4.296 | 15 | 0.0012 (adjusted) |
| Figure S2F | KA vs KA + B9 | Dunnett’s multiple comparisons test | q = 2.921 | 15 | 0.0195 (adjusted) |
| Figure S2H | Overall comparison among three groups | Ordinary one-way ANOVA | F = 15.21 | 2, 6 | 0.0045 |
| Figure S2H | KA vs Ctrl | Dunnett’s multiple comparisons test | q = 5.342 | 6 | 0.0032 (adjusted) |
| Figure S2H | KA vs KA + B9 | Dunnett’s multiple comparisons test | q = 3.858 | 6 | 0.0149 (adjusted) |
| Figure S2I | Overall comparison among three groups | Ordinary one-way ANOVA | F = 19.92 | 2, 6 | 0.0022 |
| Figure S2I | KA vs Ctrl | Dunnett’s multiple comparisons test | q = 6.045 | 6 | 0.0017 (adjusted) |
| Figure S2I | KA vs KA + B9 | Dunnett’s multiple comparisons test | q = 4.594 | 6 | 0.0067 (adjusted) |

All t tests were two-sided. Numerical P values are reported as provided by Prism; values displayed by Prism only as <0.0001 or >0.9999 are retained in that form. For ANOVA-based analyses, the omnibus test statistic and numerator and denominator degrees of freedom are reported, together with the test statistic, degrees of freedom, and multiplicity-adjusted P value for each post hoc comparison.

**References**

1.	Yang Q, Huang Z, Luo Y, Zheng F, Hu Y, Liu H, et al. Inhibition of Nwd1 activity attenuates neuronal hyperexcitability and GluN2B phosphorylation in the hippocampus. EBioMedicine. 2019;47:470–83.
